# Bond softness sensitive bond-valence parameters for crystal structure plausibility tests

**DOI:** 10.1107/S2052252517010211

**Published:** 2017-08-02

**Authors:** Haomin Chen, Stefan Adams

**Affiliations:** a Department of Materials Science and Engineering, National University of Singapore, 9 Engineering Drive 1, Singapore 117575, Singapore

**Keywords:** bond-valence method, coordination numbers, crystal radii, bond-valence parameters, bond softness

## Abstract

Based on a description of bond valence as a function of valence electron density, a systematic bond softness sensitive approach to determine bond-valence parameters and related quantities such as coordination numbers is elaborated and applied to determine bond-valence parameters for 706 cation–anion pairs. While the approach is closely related to the earlier *softBV* parameter set, the new *softNC1* parameters proposed in this work may be simpler to apply in plausibility checks of crystal structures, as they follow the first coordination shell convention. The performance of this *softNC1* bond-valence parameter set is compared with that of the previously derived *softBV* parameter set that also factors in contributions from higher coordination shells, and with a benchmarking parameter set that has been optimized following the conventional choice of a universal value of the bond-valence parameter *b*. The results show that a systematic adaptation of the bond-valence parameters to the bond softness leads to a significant improvement in the bond-valence parameters, particularly for bonds involving soft anions, and is safer than individual free refinements of both *R*_0_ and *b* from a limited number of reference cation environments.

## Abstract 2

Consistent sets of bond-valence parameters comprising 706 types of cation–anion pairs are derived and evaluated with respect to the impact of variable bond softness *b*, the first coordination shell convention and an unbiased determination of the cation coordination number.

## 1. Introduction   

### 1.1. Motivation and objective   

Empirical relationships between the length *R*_*M*—*X*_ of a bond between a cation *M* and an anion *X* and its bond valence *s*,
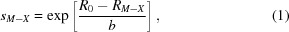
are widely used in crystal chemistry to identify plausible equilibrium sites for an atom as those sites for which the bond-valence sum (BVS) of the atom matches the modulus of its oxidation state. Following Brown & Altermatt (1985 ▸), conventionally only interactions in the first coordination shell are considered as contributing to the BVS of a cation. In our earlier work, we suggested a systematic adjustment of bond-valence parameters to the bond softness (Adams, 2001 ▸; Adams & Swenson, 2002 ▸; Brown, 2009 ▸) and published the *softBV* parameter set that implements a systematic variation of the softness parameter *b* along with *R*_0_ that also factors in interactions with counterions in higher coordination shells (Adams, 2014 ▸). More recently, other authors have also proposed sets of bond-valence parameters with flexible bond-valence parameters *R*_0_ and *b*, most notably Gagné & Hawthorne (2015 ▸).

In this context, the decision on whether or not to include weak interactions to more distant counterions beyond the first coordination shell in the determination of bond-valence parameters mostly depends on the purpose of the BVS calculations. While for the modelling of ion transport pathways as regions of low bond-valence mismatch or low bond-valence site energies, a self-consistent cut-off that prevents artefacts at the boundary between coordination shells is required (Adams, 2001 ▸), the computationally simpler first coordination shell cut-off criterion might in many cases be sufficient when the purpose is just to check the plausibility of a crystal structure, where the atoms can be expected to be located at local minima of the BVS mismatch.

From the point of view of identifying the appropriate bond-valence parameters *R*_0_ and *b* the conventional first coordination shell approach, however, entails a major problem: it seriously limits the range of interaction lengths that occur in reference structure data sets, which not only affects the value of the bond-valence parameter *R*_0_ [*i.e.* the distance corresponding to an individual bond valence of 1 valence unit (v.u.), which should not be mistaken for a typical bond distance], it also makes it more difficult (or even fundamentally impossible in the case of cations that only occur in one type of high-symmetry coordination) to determine the appropriate value of the bond-valence parameter *b*. Moreover, limiting the interactions to the first coordination shell also involves the issue that this limit of the coordination shell has to be determined in a systematic and unbiased way, because inconsistent or systematically biased choices of the coordination shell boundaries may cause significant inaccuracies. This is particularly a problem when compilations of bond-valence parameters from different sources using different definitions of the first coordination shell have to be used. Thus it becomes desirable (i) to derive a comprehensive bond-valence parameter set using a consistent approach, (ii) to derive a rational and consistent approach for deciding up to which cut-off distance a cation–anion interaction should be included in the BVS and (iii) to incorporate additional safe information when deciding on the bond softness parameter *b*.

In this work we therefore derive and investigate a new simpler way of calculating a bond softness sensitive parameter set named *softNC1*, where we refine only *R*_0_ using the first coordination shell approach and combine it with the unchanged value of *b* that we previously found for the *softBV* parameter set, where including contributions from higher coordination shells allowed for a sufficiently wide range of interaction distances and in many cases allowed for an unbiased determination of individual *b* values, and therefrom revealed a systematic correlation of the bond softness with the absolute softnesses of the interacting ions. At the same time, we aim to establish guidelines for further bond-valence parameter set determinations. The quality of the predictions resulting from the new *softNC1* parameter set is then compared with both the full slightly updated *softBV* parameter set and with a ‘conventional’ parameter set that follows the traditional approaches of a universal choice of *b* = 0.37 Å which considers only interactions in the first coordination shell. The new parameter set will also allow a quantitative judgement as to whether factoring in differences in bond softnesses *via* a systematic adjustment of *b* values remains advantageous even when simplifying the BV calculations by considering only the interactions within the first coordination shell.

### 1.2. An electron-density functional approach to bond valence   

Before we discuss our redetermination of bond-valence parameters, it is appropriate to give a brief summary of the rationale of why it appears justifiable to assume that the most suitable bond-valence parameters *R*_0_ and *b* for a cation–anion (or more strictly speaking Lewis acid–Lewis base) pair should – in principle – be predictable *a priori*. A more detailed discussion of this aspect can be found in our recent work (Adams, 2014 ▸): when two atoms approach each other from a large distance, the fraction of the atom pair’s integral electron densities that is located in the bonding region, and hence the strength of the interaction, will increase. It is thus straightforward to explore the link between bond valence and electron density. While within an atomic core the electron density, ρ(*r*), is a complex orientation-dependent function of the distance *r* from its centre, for the longer range distances relevant to interatomic interactions it will obey an exponential decay function
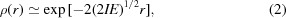
where the ionization energy *IE* of the atom controls the decay in electron density (Morrell *et al.*, 1975 ▸). Based on this observation, the concepts of bond path (BP) and bond critical point (BCP) have been worked out in Bader’s ‘quantum theory of atoms in molecules’ (see *e.g.* Bader, 1990 ▸; Weinhold, 2012 ▸). The electron density descends steeply along a bond path BP(*r*) from the atom core towards a stationary point, the BCP. The electron density at the BCP, 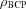, as well as its Laplacian, 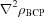, are experimental observables accessible from X-ray diffraction and may also be calculated *ab initio* as reference points for rationalizing BV parameter choices.

If, in a zero-order approximation, we assume that the electron density at a point *r* along the bond path between two atoms *M* and *X* at a distance *R*_*M*—*X*_ arises from the linear combination of otherwise unchanged electron densities ρ(*r*) = 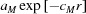 + 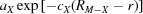, then the total electron density will assume a minimum along the bond path at the BCP and this electron density 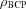 may, with the substitutions
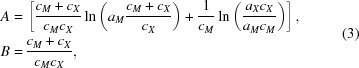
be expressed in the functional form
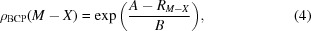
which emphasizes the close formal analogy between 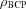 and the bond valence *s*_*M*—*X*_.

Since the coefficients *c*_*M*_ and *c*_*X*_ in equation (3) are, according to equation (2), just functions of the respective ionization energies, it becomes obvious that the denominator *B* = 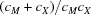 will, for a fixed average ionization energy, increase with increasing difference in ionization energies. In other words, the denominator *B* will already in this oversimplified model be a function of the electronic softnesses of both atoms. It is plausible to expect that, for Lewis acid–Lewis base type interactions, the perturbation in electron density at the BCP by the so-far neglected interaction of the electron densities will affect the values of the parameters *A* and *B* but leave the functional form of the correlation unchanged, so that the simple power-law relationship
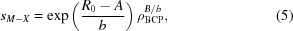
between the valence electron density at the BCP and the bond valence should be preserved. Indeed, we recently demonstrated such a close power-law relationship of *softBV* bond-valence values using literature data for 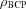 for 303 *M*—O^2−^ bonds (from Downs *et al.*, 2002 ▸) and 108 *M*—S^2−^ bonds (from Gibbs *et al.*, 1999 ▸). Analogously, the bond valence may also be expressed as a function of the Laplacian 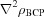, atomic hardness or electronegativity difference and atomic row number (Adams, 2014 ▸). The functional relationships between bond valence and electron density at the BCP generally involve a scaling based on the principal quantum number of the atoms involved or a closely correlated quantity (such as mass, atomic number *etc*.) and, at least for the Laplacian, obviously a measure of atomic polarizability (such as the atomic hardness or its inverse the atomic softness). This is the underlying reason why bond softness, defined as the difference between the absolute softnesses of interacting ions, should be taken into account when deriving bond-valence parameters.

### 1.3. Practical identification of bond softness-adapted *b* values   

Approximating the bond-valence parameter *b* (which represents the softness or compliance of a bond to external forces) by a universal value reduces the structural information from an approximation that takes into account both structure type and atomic properties to a cruder estimate based solely on coordination number. Improving the estimate by retaining the information on the influence of atomic properties primarily requires an independent measure of ‘bond softness’ from experimentally or *ab initio* computationally accessible quantities. Parr & Pearson (1983 ▸) proposed the characterization of individual particles in equilibrium by their constant site-independent electronic chemical potential μ
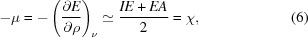
and the global average of the (site-dependent) absolute hardness η
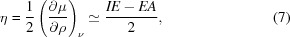
or its inverse the absolute softness σ = η^−1^. Again, ρ represents the electron density, while the subscript ν indicates the potential of the nucleus and external influences. In this approximation −μ corresponds to the absolute Mulliken electronegativity χ. The approximate identification with the independently accessible ionization energy *IE* and electron affinity *EA* values was originally derived for neutral particles, but according to Pearson (1985 ▸) the electronegativities and hardnesses of *M* ^*m*+^ cations may be calculated analogously using the (*m*+1)th ionization energy of *M* as *IE* and replacing *EA* by the *m*th ionization energy. For anions, Pearson suggested using the values of *IE* and *EA* for the neutral elements as a rough approximation. As shown in our earlier work (Adams & Rao, 2009 ▸; Adams, 2014 ▸), an empirical correlation between anion radius and anion softness may be utilized to obtain a more precise estimate: to eliminate a shift in the softness *versus* radius relationships for halides and chalcogenides, we use – in line with Pearson’s suggestion – the softness values of neutral atoms for the monovalent anions, but reduce the softnesses of the divalent chalcogenide anions by 0.017 eV^−1^. The true anion softness values will still be slightly overestimated by this approximation, but our modified softness definition appears sufficient at least to achieve comparability among chalcogenide and halide anions.

**Figure 1:**
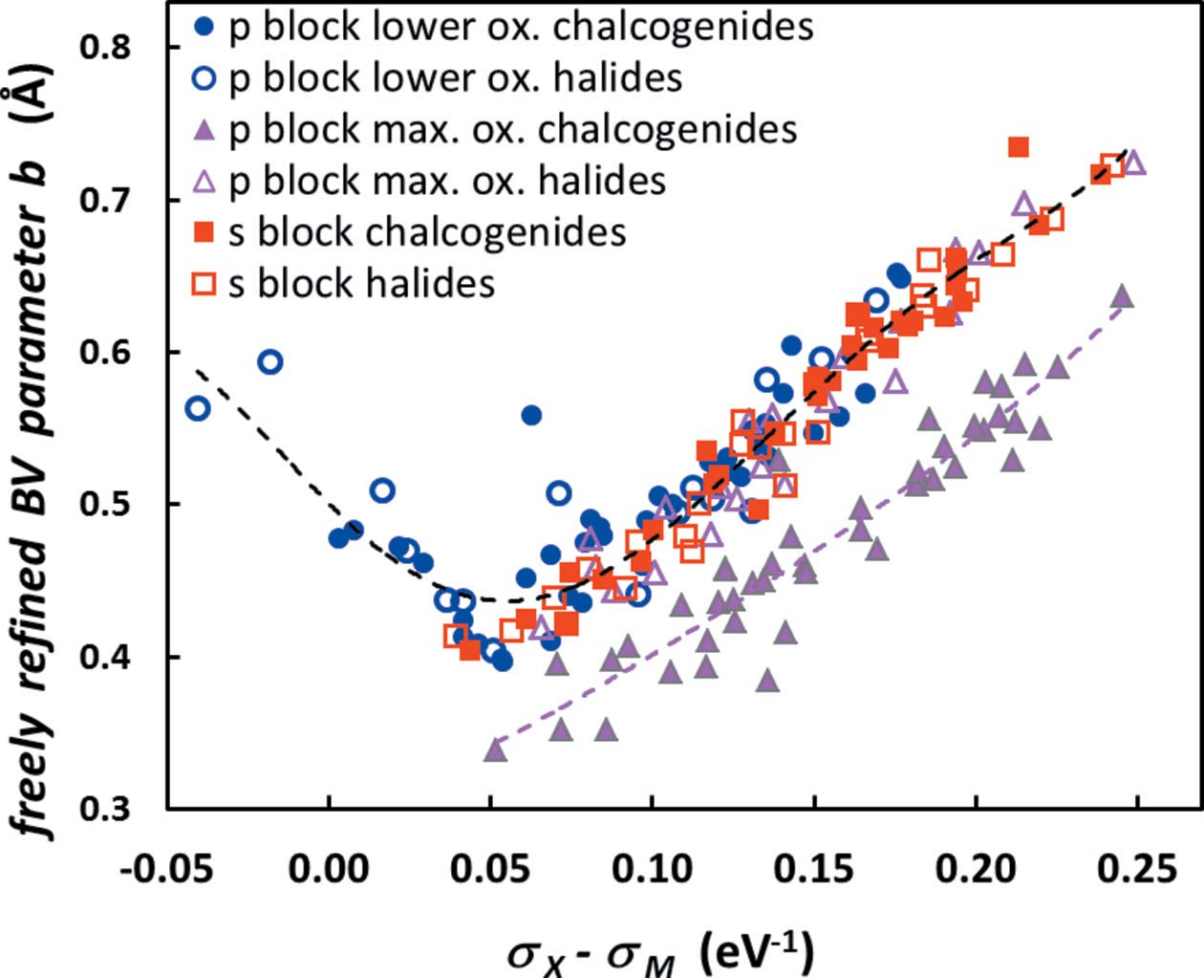
Correlation of freely refined *b* values for main group cations in interactions with halide (open symbols) or chalcogenide anions (filled symbols). The dashed black line represents a polynomial fit to the data, except for the chalcogenides of *p* block cations in their maximum oxidation state (filled mauve triangles). For the latter, a polynomial fit (dashed mauve line) yields a parallel trend with somewhat lower *b* values (redrawn after Adams, 2014 ▸).

Pearson’s empirical hard and soft acids and bases (HSAB) concept implies that reactions occur most readily between species of matching softness, which should lead to steeper interatomic potentials for these bonds and consequently to a relatively small value of the bond valence *b* compared with the *b* values for the weaker bonds between particles of mis­matched softnesses. Thus, in preparation for the determination of the *softBV* parameter set, we conducted comprehensive free refinements of bond-valence parameters. As seen in Fig. 1 ▸, the lowest *b* values are actually found for softness differences of *ca* 0.05 eV^−1^, whereas for cation–anion pairs with higher softness differences (as well as for the limited number of pairs with smaller or even negative softness differences) progressively higher values of *b* were found. The apparent shift of the minimum to positive softness differences may be tentatively attributed to the above-mentioned systematic overestimation of anion softness.

For main group cations (with the exception of *p* block cations in their maximum oxidation state in bonds to chalcogenides) the fitted *b* values (in ångström) can be approximated as a function of the softness difference σ_*X*_ − σ_*M*_ (in eV^−1^) by the fifth-order polynomial *b* = 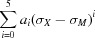, shown as a black line in Fig. 1 ▸ with the coefficients *a*_5_ = 2479.6 Å eV^5^, *a*_4_ = −1384.2 Å eV^4^, *a*_3_ = 198.75 Å eV^3^, *a*_2_ = 10.428 Å eV^2^, *a*_1_ = −2.1316 Å eV and *a*_0_ = 0.5009 Å. For *p* block cations in their maximum oxidation state in bonds to chalcogenides, a simpler second-order polynomial fit with *a*_2_ = 1.9108 Å eV^2^, *a*_1_ = 0.8287 Å eV and *a*_0_ = 0.2946 Å was used to predict the systematically lower *b* values, since the softness difference for all observed cases was 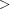
0.05 eV^−1^. Analogous polynomial fits based on the set of reference data available at that time have been used to derive the systematic *b* values in the *softBV* parameter set.

The *b* values of the bond softness sensitive BV parameter set derived in the way sketched above are somewhat larger than the ‘universal value’ of 0.37 Å. The difference will be affected by the bias towards small *b* values that is introduced when weak interactions from higher coordination shells are ignored, and hence a free refinement of *b* values (where reliably possible) would be expected to reduce the fitted *b* values slightly. Here, for the purposes of the proposed new parameter (obeying the first coordination shell convention) we prefer to retain the same *b* parameters, largely because their determination appears more reliable and thus they should be a more appropriate measure of the true bond softness. As demonstrated in our earlier work (Adams, 2014 ▸), the correlation coefficient of the fundamental *s*(ρ_BCP_) relationship is higher when *s* is calculated from the softness sensitive *softBV* using these *b* values than for conventional bond-valence data relying on a fixed value of *b*.

## 2. Objective and computational methods   

In this work we have determined consistent sets of bond-valence parameters comprising, besides *R*_0_ and *b*, the cut-off distance *R*_cutoff_ and the average coordination numbers *N*_C_ for 706 cation–anion pairs using three different conventions based on the same reference data set containing (after the necessary elimination of outliers) 15 523 reliable cation environments:

(i) Softness sensitive variable *b* values adapted from the *softBV* parameter set (Adams, 2001 ▸) factoring in effects of higher coordination shells. In this case, we include interactions beyond the first coordination shell up to a cut-off distance 4 Å < *R*_cutoff_ < 8.5 Å. The results can be understood as a slightly updated version of our previously published *softBV* parameter set.

(ii) A new *softNC1* parameter set that retains the same softness sensitive *b* values as we found for the *softBV* parameter set, but constraining the cut-off distance to the boundary of the first coordination shell *R*_cutoff_ = *R*_1_ as the basis for revised fits of *R*_0_ values. This also involves deriving and testing a method for a systematic determination of the limits of the first coordination shell.

(iii) For benchmarking purposes, a ‘conventional’ BV parameter set *convBV* has also been determined. In other words, we fitted *R*_0_ values for our reference data set based on the conventional choices of a fixed universal value of *b* = 0.37 Å and, as for the second parameter set, refined *R*_0_ values under the assumption that only counterions from the first coordination shell contribute to the BVS.

### 2.1. Selection of the set of reference crystal structures   

The determination of BV parameters typically requires, as the first step, the compilation of a database of reliable reference crystal structure data. In our work the main source is the Inorganic Crystal Structure Database (ICSD) (Bergerhoff & Brown, 1987 ▸), complemented by structures extracted from the recent literature. The guidelines for our selection of compounds have been that the reference structures:

(i) Must have been experimentally determined by X-ray or neutron diffraction with reasonably low residuals *R*_csr_ of the crystal structure refinement, rather than structures predicted computationally. *R*_csr_ is chosen here instead of the common term ‘*R* value’, to prevent confusion with bond lengths *R_M—X_*. Where the database and literature comprise a sufficient number of available cation environments, we aimed at *R*_csr_ values ≤ 0.055, but compromises were made for cation–anion pairs with fewer available data.

Although for a given crystal structure a smaller *R*_csr_ value should indicate a more reliable structure model, inconsistencies in the type of *R*_csr_ values reported in databases, as well as the small influence of light atoms on *R*_csr_ values from X-ray diffraction data, limit its significance and so it should not be used as the only criterion.

Reference structures for H^+^–anion bonds are based exclusively on neutron diffraction data due to the systematic underestimation of bond lengths to H^+^ in X-ray structure determinations.

(ii) Must contain only one type of anion, namely the type to be determined.

(iii) Must have been determined at or near room temperature and at ambient pressure.

(iv) Should not include any sites with partial or mixed site occupancy. This also rules out structures where an ion has a non-integer oxidation state (which may be thought of as equivalent to the mixed occupation of a site by the same element in two different oxidation states).

(v) Should not contain metallic bonds (among anions or among cations) or involve an atom with zero oxidation state.

(vi) Should preferably contain at least two types of cation, including the type to be determined, and should not contain H^+^ (except when H^+^ is the cation of interest). On the other hand, it is also advisable to limit the complexity of reference structures, so that in practice we tried to focus on compounds with two or three types of cation and one type of anion as reference structures.

(vii) Structure models for modulated structures were excluded, as they are often of limited precision and would – if considered – bias the reference data sets by the typically numerous inherently similar cation environments that a single structure contains.

(viii) If a sufficient number of reference structures fulfilling the above criteria were available for a cation–anion pair, the number of reference structures of the same structure type and the number of reference structures with the cation in high-symmetry environments were limited. Including multiple structure refinements of the same compound (*e.g.* a compound of high technological or scientific relevance) was generally avoided. For a number of parameters involving the H^+^ cation, no structures satisfying all requirements could be identified from the ICSD. In such cases, requirement (iv) was lifted, which will lead to a lower dependability of these parameters.

**Figure 2:**
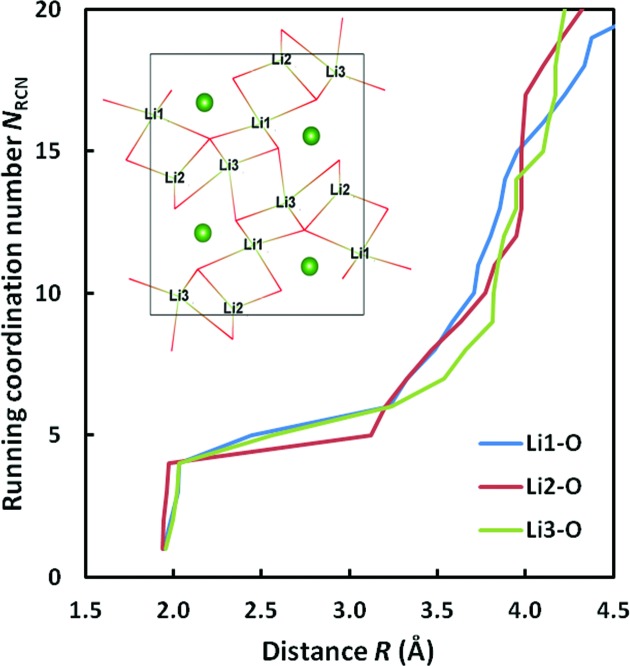
The structure of Li_3_BO_3_ supplies three different environments to the Li^+^—O^2−^ bond-valence parameter determination, marked as Li1, Li2 and Li3.

After the identification of reference structures for the determination of bond-valence parameters between a cation *M* ^*m*+^ and anion *X* ^*x*−^, a number of cation environments were extracted from these structure data. ‘Environment’ here refers to a list of distances between the particular cation of interest *M* ^*m*+^ and all surrounding *X* ^*x*−^ anions in the structure up to a sufficiently high distance (5–9 Å). Each structure may contain several distinct environments for distinct *M* ^*m*+^ cations. For example, the structure of Li_3_BO_3_ sketched in Fig. 2 ▸ contains three distinct Li^+^ environments that can be considered in the determination of the bond-valence parameters for Li^+^—O^2−^. Each environment will carry the same weight for its BVS during the refinement of bond-valence parameters. This may not be optimal as such environments are, strictly speaking, not independent observations but correlated *via* structures, but we currently have no convincing method for assigning different weights to different environments. In some cases, where a single low-symmetry compound contained a large number of symmetrically distinct yet similar environments that would have dominated the parameter refinement, we chose to reduce the number of these environments that were considered in the refinement (arbitrarily giving preference to the cation that had been given the lower number in the database entry).

### 2.2. Bond-valence parameter refinement approach   

For the case of the *softBV* parameters, in principle both bond-valence parameters, *b* and *R*_0_, have to be determined. One possible way is to fit *b* together with *R*_0_. The minimization process must then ensure that the refined parameters:

(i) Yield a zero average mismatch of the cation BVS for the reference structure data set, 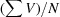 = *V*_id_ [where *V*_id_(*M*) is the oxidation state of cation *M*], and at the same time

(ii) Minimize the biased standard deviation Δ*V* = 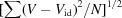 of the cation BVS.

This refinement process may involve the need to eliminate outliers that would strongly bias the refined parameters. Still, for each such environment flagged as an outlier we tried to evaluate whether there are further lines of evidence suggesting a problem with the underlying structure refinement and checked that the elimination does not unduly bias the balance between different coordination numbers in the surviving reference data set.

This approach (which was used to determine the data points in Fig. 1 ▸) reveals the underlying trends but results in a significant scatter of parameter values if the number of available cation environments is too low, does not contain sufficiently different coordination types or is highly vulnerable to undetected erroneous cation environments. We therefore follow the approach chosen in our *softBV* parameter set to reduce the scatter in the refined *b* values by utilizing the systematic trends observed in Fig. 1 ▸. In line with our earlier work, *b* values for halides and chalcogenides (where anion softness could be refined) are assigned employing the polynomial fits derived in Section 1.3 from the free refinements based on the difference between the softnesses of the anions and cations involved. For pnictide anions (N^3−^, P^3−^, As^3−^, Sb^3−^) and for H^−^, the lack of available reliable anion softness values motivated us to retain the freely refined values *b*.

After the derivation of systematic bond softness dependent *b* values and retaining the corresponding cut-off distances *R*_cutoff_ for interactions from higher coordination shells (that were chosen so that a reduction in *R*_cutoff_ by 1 Å did not reduce the BVS by more than 1% when *R*_0_ and *b* were kept fixed), *R*_0_ values were finally redetermined. The results are essentially identical to the previously published *softBV* parameters except for minor updates to the list of cation–anion pairs and reference structures. Since *R*_0_ is the only free variable, the refinement procedure is simplified here to:

(i) Read the *b* value that corresponds to the softness difference between cation and anion.

(ii) Choose an initial value of *R*_0_.

(iii) Vary *R*_0_ iteratively so that the BVS averaged over all cation environments matches the oxidation state of the cation.

In principle, the refinement procedure for the two alternative parameter sets *softNC1* (with *softBV* BV values but the limit of the first coordination shell as cut-off distance) and the *convBV* benchmarking set (with fixed *b* = 0.37 Å and first coordination shell as cut-off distance) may appear analogous. Still, to refine a set of bond-valence parameters limited to the first coordination shell, the (*a priori* unknown) radius of this first coordination shell *R*_1_ must be refined concurrently with other parameters, as it functions as the cut-off distance *R*_cutoff_ for the interactions to be taken into account. Hence we will briefly discuss the determination of *R*_1_ and the associated determination of the coordination number in the following section.

### 2.3. Coordination number and boundary of the first coordination shell   

**Figure 3:**
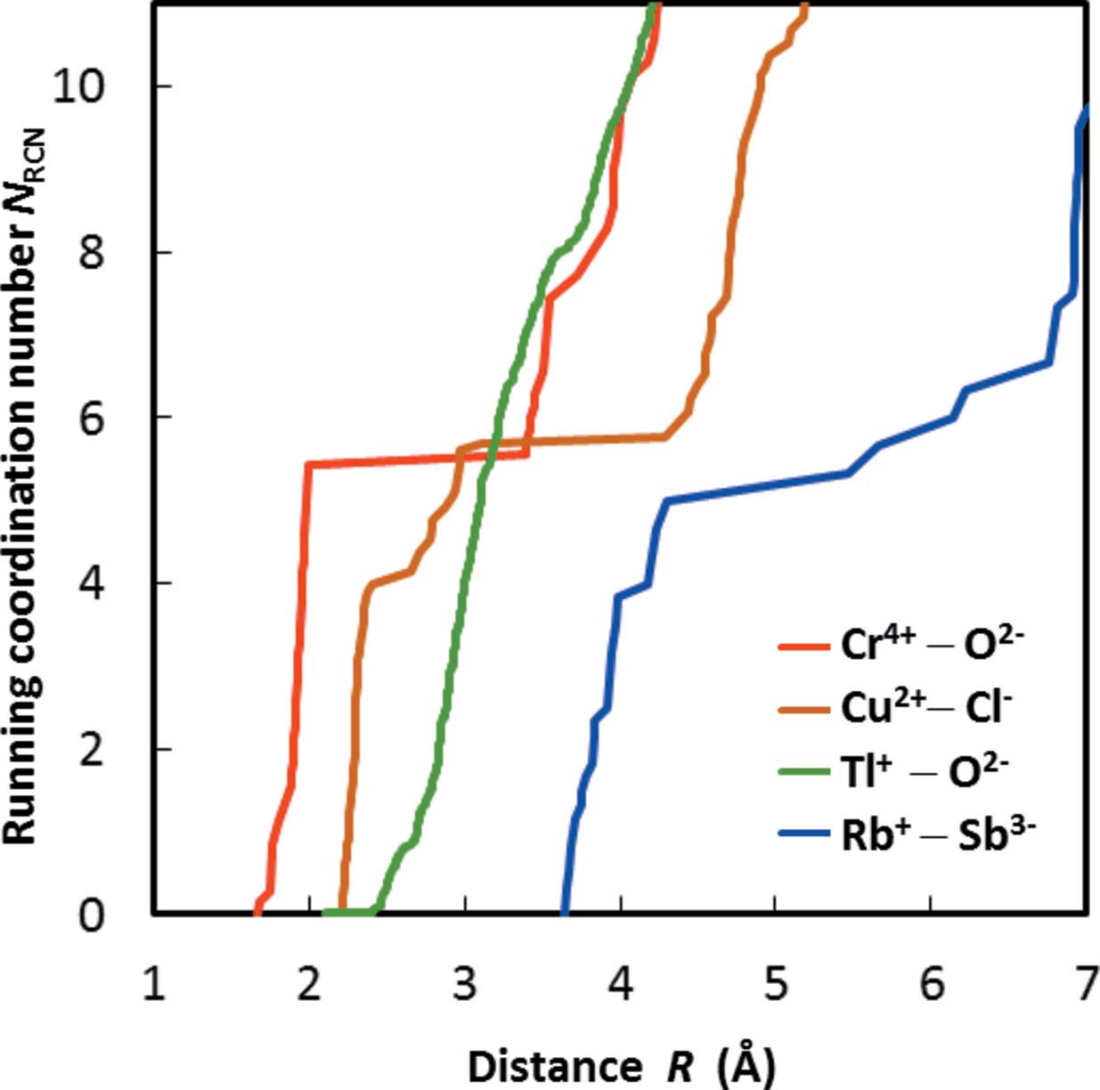
Selected examples for the variation of the running coordination number *N*_RCN_ with *R* in reference data sets. Cu^2+^—Cl^−^: two plateaux within the first coordination shell due to prevalent (4+2) coordination. Rb^+^—Sb^3−^: two plateaux due to subsets of varying *N*_C_ from 4 to 6 in the reference data. Cr^4+^—O^2−^: one single plateau. Tl^+^—O^2−^: no obvious plateau.

In a glass or liquid the running coordination number *N*_RCN_ *versus* radius *R*, as well as its (scaled) gradient, the radial distribution function *g*(*R*), can be expected to be continuous. The first local minimum of the radial distribution function then defines the cut-off *R*_1_ for the first coordination shell. Similarly, identifying the boundaries of the first coordination shell for an individual cation environment in a crystal structure is straightforward, as long as the first and second co­ordination shells are separated by a clear plateau in the corresponding *N*_RCN_(*R*) graph. However, when determining the values of *N*_C_ and *R*_1_ systematically from reference data sets containing a limited number of cation environments, there are often neither sufficient data points to fit a smooth curve and therefrom *R*_1_ unambiguously as the first minimum of *g*, nor clear and sufficiently wide plateaux distinguishable. Instead, the necessary inclusion of cation environments with different anion arrangements and coordination numbers, as well as electronic distortions of transition metal cation environ­ments, can produce various complex shapes of *N*_RCN_(*R*) plots, among which a few representatives are selected in Fig. 3 ▸. There are cases like Cr^4+^—O^2−^ which demonstrate a clear plateau that resembles a single-crystal environment, and cases like Tl^+^—O^2−^ whose *N*_RCN_ curves are continuous as in a liquid environment. There are also a large number of cases showing multiple platueax. In the Rb^+^—Sb^3−^ data set, individual environments are of coordination numbers 4, 5 and 6 that give rise to two plateaux. In the case of Cu^2+^—Cl^−^, most of the environments show a (4+2) coordination configuration, where the two anions are present between the first and second coordination shells.

In order to render a consistent and automatic determination of the first coordination shell possible under such varying circumstances, the values of *N*_C_ and *R*_1_ for a cation–anion pair *M*—*X* were determined iteratively according to the following formula whenever *R*_0_ was varied during the refinement:
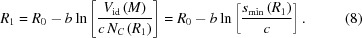
As will be discussed below, this defines the limit of the first coordination shell as the distance *R*_1_ for which the bond valence *s*(*R*_1_) equals the fraction 1/*c* of the bond valence for the typical bond distance *R*_min_ within the first coordination shell.

For the refinement of the *softNC1* parameter set, we used *softBV* values for *b* and *R*_0_ as the initial values, while *N*_C_, *R*_1_ and *R*_0_ were refined simultaneously using the following procedure:

(i) Starting from initial guesses of *R*_0_ and *N*_C_, calculate *R*_1_.

(ii) Until *R*_0_, *N*_C_ and *R*_1_ all converge, do the following iteratively:

(*a*) Search for *R*_0_ so that 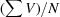 = *V*_id_.

(*b*) Calculate *N*_C_ as the number of anions present within *R*_1_.

(*c*) Calculate *R*_1_ using equation (8).

(iii) Record final values of *R*_0_, *N*_C_ and *R*_1_ and calculate the standard deviation Δ*V* for the data set with these refined parameters.

Since *N*_C_ is not known *a priori*, the calculations are conducted for a wide range of possible initial values of *N*_C_ from 2 to 20 with an increment of 1, and among the resulting 19 sets of refined *R*_1_, *R*_0_ and *N*_C_ values, the set that corresponds to the smallest Δ*V* in BVS is accepted. In most cases, the refinement results turned out to be the same for a plausible range of initial choices of *N*_C_.

At this point the only remaining issue was to identify a plausible value for the factor *c* in equation (8). Again, we avoided imposing a predefined value and tested a number of possible values 1 ≤ *c* ≤ 8. After initial checks it turned out that the range of plausible values for *c* can be narrowed down to 3 ≤ *c* ≤ 6, as these choices lead to consistent results for most cation–anion pairs. To establish a more precise value of *c* we visually inspected the *N*_RCN_(*R*) curves for all 88 cases for which the refined values *N*_C_(*c* = 3) and *N*_C_(*c* = 6) differed by ≥ 0.5 to decide which of the choices of *c* yields the most plausible value of *N*_C_.

## 3. Results and discussion   

### 3.1. Bond-valence parameter lists   

The refined bond-valence parameter values *R*_0_ and *b* for all refined parameter sets for 706 cation–anion pairs are reported in Table S1 in the supporting information, along with the respective cut-off distances *R*_cutoff_ and the average coordination numbers *N*_C_ Besides refining these values in a consistent framework for use in plausibility checks of crystal structures, our main objective has been to analyse whether and to what extent the two separate simplifications in the parameter refinement, namely eliminating the effect of higher coordination shells in the *softNC1* and *convBV* sets and additionally fixing the value of *b* in *convBV* to 0.37 Å, will affect the quality of predictions, specifically for their application in crystal structure plausibility tests.

### 3.2. Coordination numbers and cut-off distance   

**Figure 4:**
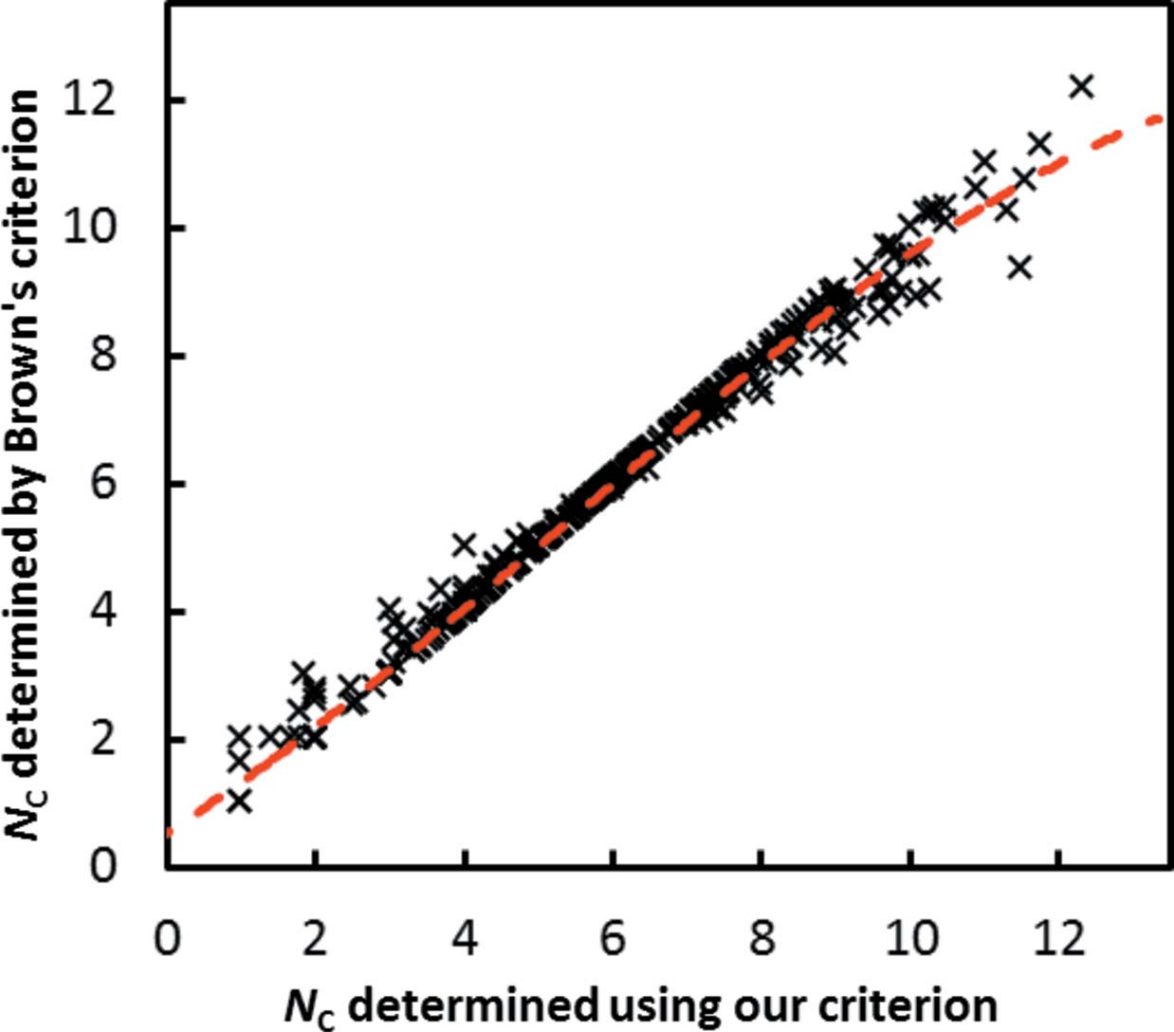
Comparison of coordination numbers *N*_C_ determined using Brown’s criterion and the criterion ultimately proposed in this work.

For the *softNC1* and *convBV* parameter sets that factor in only the interactions in the first coordination shell, it was necessary as the first step to achieve a systematic determination of the coordination number *N*_C_ according to equation (8) by identifying a value of the coefficient *c* that consistently results in the correct coordination number. It may be emphasized that the value of *c* corresponds to the ratio between the bond valences at the typical bond distance *R*_min_ and at the cut-off radius *R*_1_

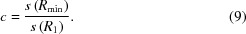
Thus, the relative spread of bond valences within the first coordination shell is fixed irrespective of the coordination number, which leads to a similar relative spread of bond lengths within the first co­ordination shell, while at the same time allowing for a slightly wider range of bond lengths for the softer bonds. In contrast, the conventional choice of any fixed bond-valence value for the limit of the first coordination shell, *e.g.* 3.8% of the cation BVS, as previously suggested by Brown & Altermatt (1985 ▸), leads to a pronounced artificial reduction in the range of bond valences (and bond lengths) that are considered as part of the first coordination shell with increasing coordination number. Consequentially, for extremely high coordination numbers the values determined using Brown’s criterion tend to be slightly too low, and slightly too high when they should be extremely low, while for the vast majority of cases both methods yield the same or closely similar values of *N*_C_, as seen from Fig. 4 ▸. In detail, the co­ordination numbers determined by both methods match exactly for 563 out of the 706 types of cation environment studied in this work, and for 655 of them they differ by less than 5%.

Using a too large or too small value of *c* might lead to calculating the coordination number on the wrong plateau for cases that show more than one plateaux (*cf*. Fig. 3 ▸). Excessively narrowing the range of permitted bond valences within the first coordination shell by using a too small *c* also tends to underestimate *N*_C_ for many cases. A quick test suggests that values of *c* 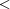 3 tend to lead to an obviously too small co­ordination number. For the obviously too small *c* = 1 (*i.e.* when limiting *R*_1_ to *R*_1_ = *R*_min_) the method would also run into convergence problems for numerous ion pairs. Similarly, for *c* ≥ 7 the resulting coordination numbers tend to grow with the choice of *c* to implausibly high values, *i.e.* such values of *c* lead to an inclusion of the second coordination shell. Thus we tested in more detail the intermediate cases *c* = 3 to 6 in steps of 0.25. Out of 706 ion pairs tested, only 88 show a variation in *N*_C_ larger than 0.5 depending on the choice of *c* over this range. The correct *N*_C_ for these entries were therefore determined by individual analysis of the *N*_RCN_(*R*) curves for 64 of the pairs, leaving out 24 cases where even visual inspection appeared inconclusive.

**Figure 5:**
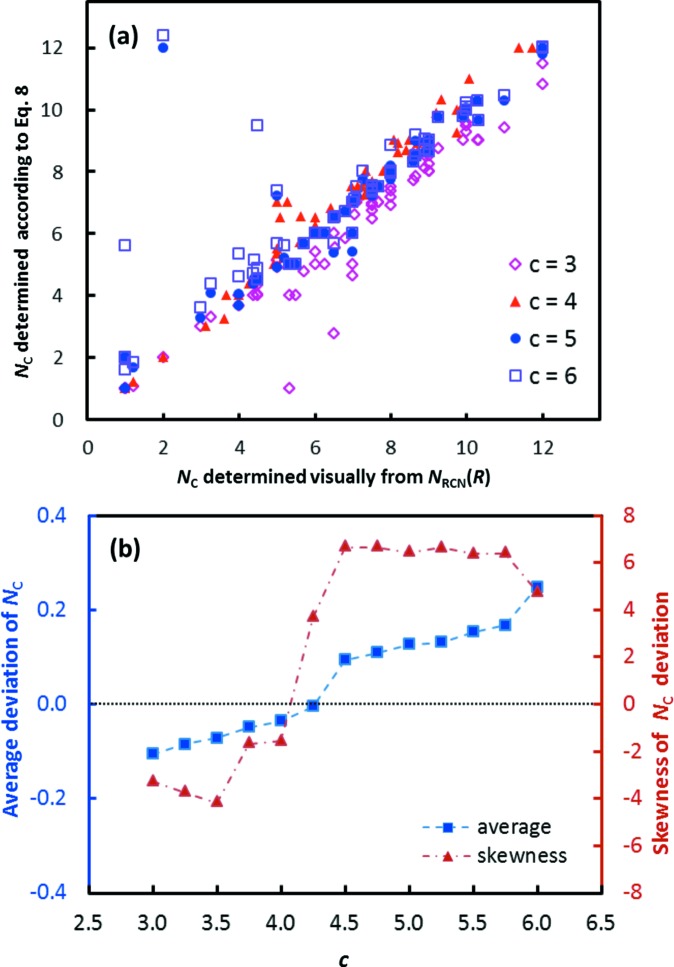
(*a*) Plot of *N*_C_ values resulting from the criterion in equation (8) for selected choices of *c* (3 ≤ *c* ≤ 6) *versus* the *N*_C_ values determined by inspection of the *N*_RCN_(*R*) curves. (*b*) Variation in average and skewness of *N_C_* deviations for *N_C_* values determined according to equation (8) relative to *N_C_* values determined visually from the *N*_RCN_(*R*) curves depending on the choice of the coefficient *c*. Both graphs refer to the analysis of the 64 cases mentioned in the text, where the proposed value of *N*_C_ is sensitive to the choice of *c*, while the visual inspection appeared unambiguous.

As seen from Fig. 5 ▸, the minimum in the deviation between the systematic determination of *N*_C_ according to equation (8) and the result of visual inspection of *N*_RCN_(*R*) occurs for *c* values around 4.25, and the same choice of *c* also minimizes the skewness of the distribution of the observed deviations within the subset of cation–anion pairs with *c*-value sensitive coordination numbers. It may be noted that Fig. 5 ▸ accentuates the deviation by omitting the majority of cases where any of the choices of *c* would lead to (nearly) the same value of *N*_C_. The final values of *R*_1_ and *N*_C_ listed in Table S1 in the supporting information for the softness-sensitive parameter sets *softNC1* are therefore based on *c* = 4.25.

The same analysis for the ‘conventional’ bond-valence parameter set (*i.e.* assuming *b* = 0.37 Å) yields a somewhat reduced dependence of the coordination number on the choice of *c*, and the smallest deviations of average coordination number and smallest skewness of the deviation distribution for the case *c* = 6. This is understandable, as the choice of a lower value of *b* means that the same distance interval between *R*_min_ and *R*_1_ will correspond to a more pronounced reduction in the bond valence.

### 3.3. Bond-valence parameters and crystal radii   

As a cross-check of our bond-valence parameters, we also compared the value of *R*_min_, *i.e.* the expected average bond distance for the average coordination number for a series of cations bonded to the same anion, with the variation in the Shannon crystal radii (Shannon, 1976 ▸)
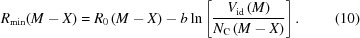
Our parameter set results in one *R*_min_ value for each cation–anion pair and the (in general fractional) average coordination number *N*_C_ for our reference data set, while the Shannon crystal radii are grouped based on integer coordination numbers *N*_C(Sh)_ for cations only. In order to compare our results, we thus need first to calculate the effective Shannon crystal radius *R*_crystal_ for the average *N*_C_ of the reference data set. For ions, where Shannon’s compilation offers multiple *N*_C(Sh)_, a linear interpolation is used to calculate the effective Shannon crystal radius at the average *N*_C_ of the reference data set. If one *N*_C(Sh)_ value was reported and the value was within ± 0.2 of our *N*_C_, the Shannon crystal radius was used without modification. The remaining 88 out of 706 cation–anion pairs with one deviating cation coordination number or without a coordination number in Shannon’s compilation were eliminated from the comparison.

**Figure 6:**
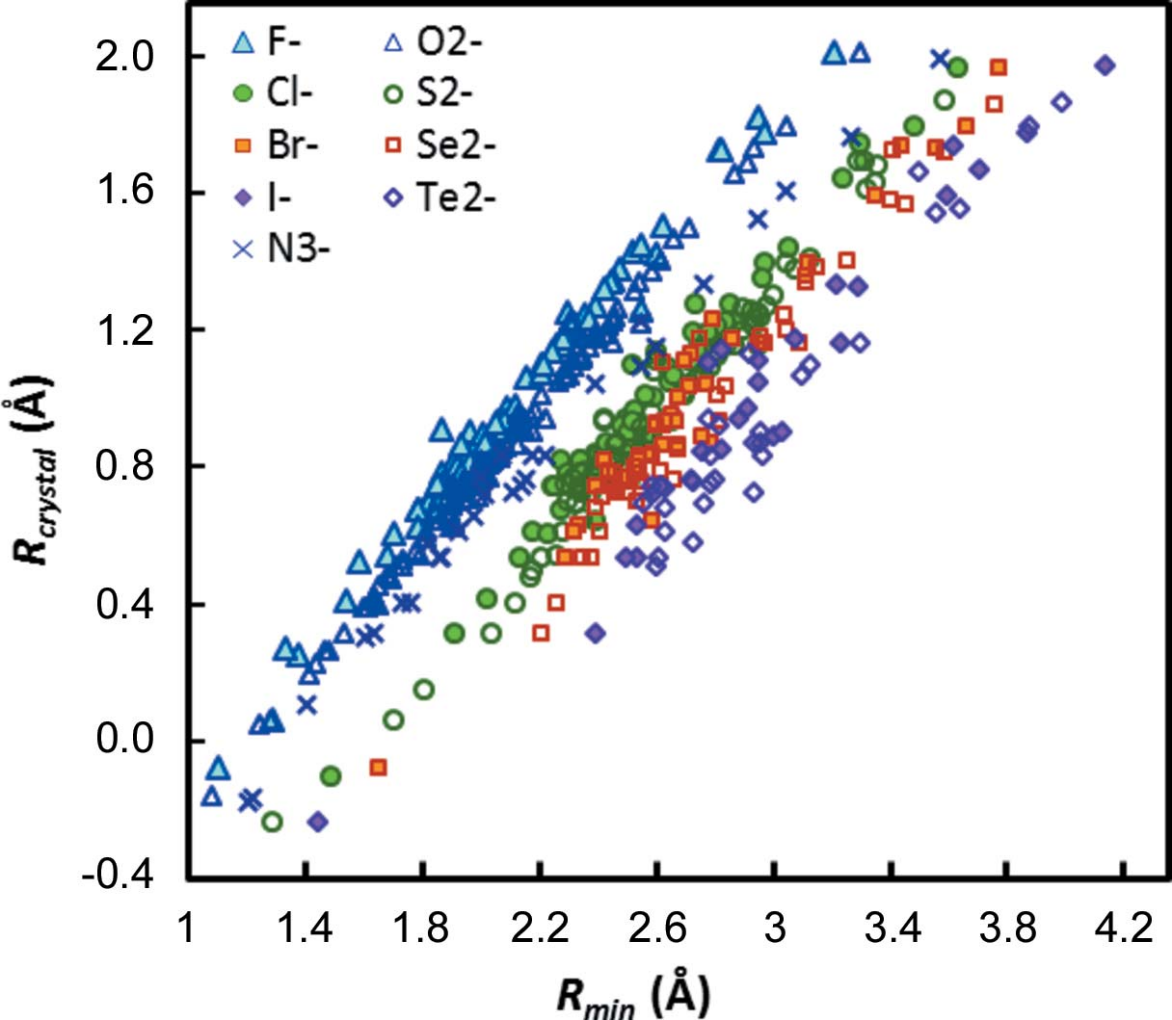
Correlation between *R*_min_ and Shannon crystal radii *R*_crystal_. The displayed *R*_min_ values are based on the softness sensitive *b* values. To reduce overlap, data are shown only for the nine anions for which more than 30 types of cation–anion pairs could be determined.

Fig. 6 ▸ shows the variation in *R*_min_ values (derived from the *softNC1* parameter set) as a function of the Shannon crystal radii *R*_crystal_ of the affected cations for selected anions. Tests for linear relationships between *R*_min_ and *R*_crystal_ for those anions, where the known anion softness allows the use of systematic *b* values, yield high correlation coefficients, *e.g. R* ^2^ = 0.994 for the correlation *R*_crystal_(*M* ^*m*+^) = 0.9901*R*_min_(*M* ^*m*+^—O^2−^) − 1.2046 Å for the 129 cation–oxide parameters shown as open triangles in Fig. 6 ▸. The corresponding relationships for other anions are listed in Table S2 in the supporting information.

While the slopes of these relationships approach unity for the harder anions O^2−^ and F^−^, slightly lower values are found for the larger softer anions (*e.g.* 0.9065 for Te^2−^ and 0.8747 for I^−^). It may be noted in passing that, by the definition of Shannon crystal radii and Shannon ionic radii, exactly the same relationships with an additional shift of −0.14 Å will apply to the Shannon ionic radii [*e.g. R*_ion_(*M*) = 0.9901*R*_min_(*M*—O) − 1.3446 Å for the oxides]. Closely similar correlations with comparable correlation coefficients are also observed when deriving the *R*_min_ values from the *convBV* data set, *i.e.* based on the conventional fixed choice of *b*. Thus, the main cause for the change in slope is that for larger and softer anions there will be a slightly more pronounced change in average coordination number for a given change in cation size.

**Figure 7:**
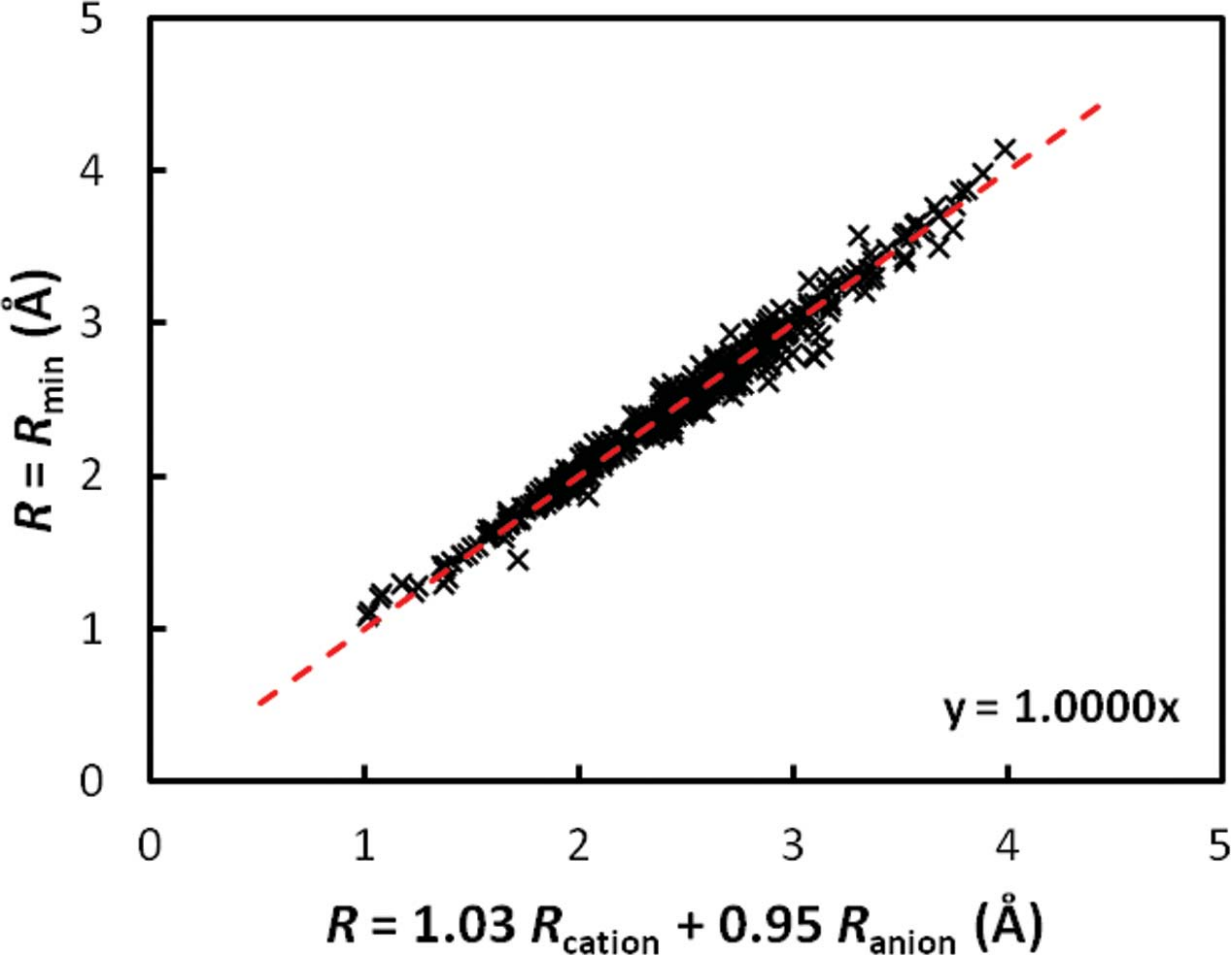
Linear correlation between *R*_min_ and the sum of the scaled Shannon radii of cations and anions.

On the other hand, the separations between different straight lines in Fig. 6 ▸ for different anions are obviously related to the respective anion sizes. Hence, linear regression with *R*_crystal,*M*_ and *R*_crystal,*X*_ as explanatory variables and *R*_min_ as response variable with or without intercept yields
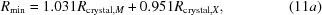
with adjusted *R* ^2^ = 0.9991, and 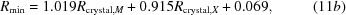
with adjusted *R* ^2^ = 0.9802. Thus, the anyway small intercept as an additional refinable parameter does not improve the agreement and can be dropped. Thereby *R*_min_ is found to correlate linearly with the sum of the slightly scaled Shannon crystal radii of cations and anions, as depicted in Fig. 7 ▸. The scaling factors 1.031 for cations and 0.951 for anions also quantify the average overestimation of anion sizes and underestimation of cation sizes by the Shannon crystal radii.

This profound correlation also allows the calculation of the missing Shannon crystal radii of P^3−^ (1.851 Å), As^3−^ (1.973 Å), Sb^3−^ (2.244 Å) and H^−^ (1.077 Å). Moreover, additional Shannon cation radii can be calculated from fitting the data shown in Fig. 6 ▸. All values are listed in Table S3 in the supporting information.

### 3.4. Comparison of parameter sets   

One of the key tasks of this project was to find out whether one of the three derived bond-valence parameter sets has a significant advantage over the other sets. To benchmark the quality of the parameter sets, we compared the average standard deviation Δ*V* of the three parameter sets.

**Figure 8:**
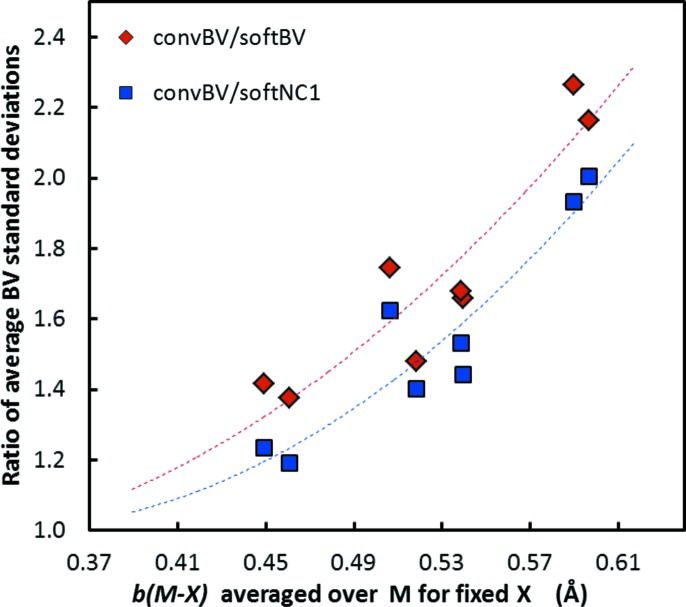
Dependence of the relative increase in standard deviations of BVSs within the reference data set when using the conventional parameter set with a fixed universal value of *b* = 0.37 Å instead of *softBV* parameters (diamonds) or *softNC1* parameters (squares) on the average *b* value for BV parameters involving the respective anion type. Data are shown for halide and chalcogenide anions only. The dashed lines are polynomial fits as a guide to the eye.

As seen from Table 1 ▸, the lowest standard deviation among the three approaches is consistently found when using the *softBV* approach. This is independent of whether all cation–anion pairs are considered, or whether the comparison involves only those parameters that can be determined with higher reliability from reference data sets containing at least 20 cation environments. When simplifying the *softBV* parameter set by considering only the interactions in the first coordination shell (while maintaining the bond softness sensitivity), there is only a small (but statistically significant) increase in the average standard deviation of the BVSs within the same set of reference cation environments. In contrast, enforcing *b* = 0.37 Å causes a much more pronounced increase in the standard deviation, *i.e.* it lowers the quality of the BVS calculations considerably. When comparing subsets of parameters with different anions (see Fig. 8 ▸), it becomes obvious that this advantage of the softness sensitive parameter sets over the conventional parameter set with a universal *b* value becomes more prominent the higher the average *b* value is for the parameters involving the respective anion. In other words, the softer the anion the more important it will become to use softness sensitive BV parameters. This is not surprising, as the original choice of *b* = 0.37 Å was suggested based on a training set consisting mainly of hard anions. It may be noted that, for oxides alone, a recent systematic study (Gagné & Hawthorne, 2015 ▸) gives an average *b* value of 0.40 Å when using the first coordination shell convention. So the value of 0.37 Å appears slightly too low even for oxides. As discussed above, the difference from the average *b* = 0.45 Å for oxides in the *softBV* parameter set is also affected by the neglect of the influence of the higher coordination shells in the conventional approach.

**Figure 9:**
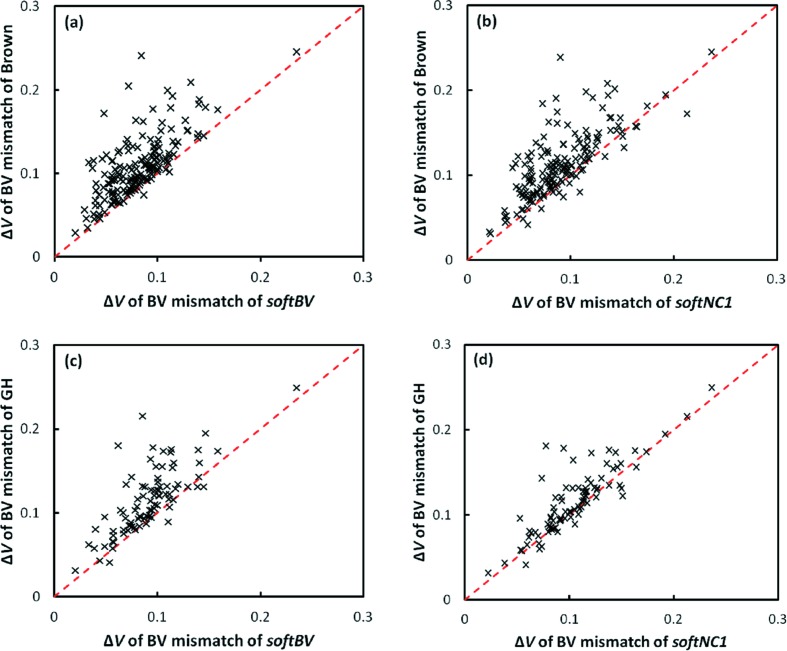
Comparison of standard deviations when using parameters from Brown’s compilation (top row) or Gagné & Hawthorne’s parameters (bottom row) with *softBV* (left-hand side) or *softNC1* (right-hand side) parameters determined in this work. The comparison involves only those cation–anion pairs with *n* ≥ 20 cation environments. The *softBV* parameter set is calculated at the higher cut-off distance suggested for this parameter set, while for the other three sets the cut-off is set to the value of *R*_1_ determined in this work as the limit of the respective first coordination shell.

We also compared our *softBV* and *softNC1* parameter sets derived in this work with Gagné & Hawthorne’s systematic determination of BV parameters (Gagné & Hawthorne, 2015 ▸) and with Brown’s compilation of BV parameters (Brown, 2016 ▸), which also contains, besides parameters from his own work, values from various other literature sources. Note that the parameters of Gagné & Hawthorne were determined by freely refining BV parameters using the first coordination shell approach. We used all four parameter sets to calculate the cation BVSs in identical reference data sets covering a wide range of oxides and compared the biased standard deviations Δ*V*. It can be seen from Fig. 9 ▸ that our *softNC1* parameter set performs better than Brown’s compilation and equally well as Gagné & Hawthorne’s data set for oxides. Our *softBV* performs better than both literature data sets, partly due to the additional inclusion of weak interactions beyond the first coordination shell.

### 3.5. When can *b* be refined freely?   

The task of refining *R*_0_ with a given fixed *b* is straight­forward, as it only involves fitting *R*_0_ so that the average BVS mismatch in the reference data set becomes zero, *i.e.* 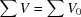 for the cation–anion pair. This is a stable process, as during the refinement a unique definite *R*_0_ for all cation–anion pairs, even those with very few compounds available, can always be reached. Refining *b* and *R*_0_ together is a more involved task, as now we must find an additional function to minimize. Conventionally, this function is taken as the biased standard deviation of BVS mismatch Δ*V* = 
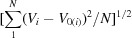
. The choice of biased standard deviation (where the sum of squares is divided by *N*) over the unbiased one (where the sum of squares is divided by *N* − 1) was made because the average BVS mismatch is known to be zero in the ideal case. It is tempting to apply this refinement and claim the generated combination of *b* and *R*_0_ as the unique ‘best fitted’ bond-valence parameters. We designed an experiment to study various factors affecting such a refinement.

**Figure 10:**
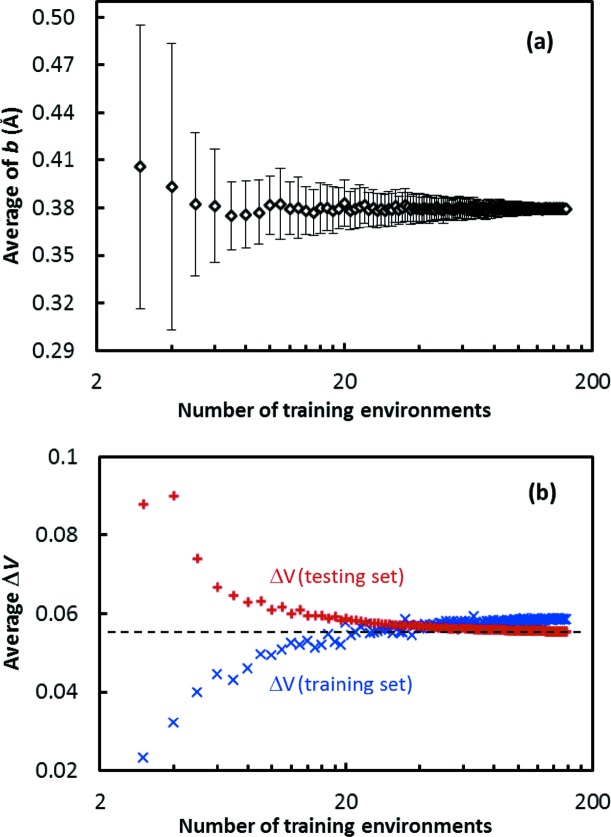
Dependence (*a*) of the average *b* value resulting from the fitting of 100 randomly chosen sets of oxide environments of B^3+^ and (*b*) of the average standard deviation in the training set and testing set on the number of B^3+^ environments in each set. The horizontal dashed line in panel (*b*) marks the true standard deviation of the complete testing set.

The B^3+^—O^2−^ pair was chosen as the subject of this study due to its large number of available environments (*n* = 315). Half of these environments were kept as the test set, for which the ‘true’ bond-valence parameters *R*_0,test_ and *b*_test_ were determined and Δ*V*_test_ calculated. The other half of the environments formed the training pool. For each *n* from 3 to 157, *n* environments were randomly selected from the training pool, from which a set of *R*_0_ and *b* were determined. This process was repeated 100 times for each *n*, and the averages of *b* and Δ*V versus n* are recorded in Fig. 10 ▸(*a*).

The ‘true’ values of *b* and Δ*V* are a function of the compounds selected in the testing set, so are bound to deviate from values converged on the training set. In order to ensure an accuracy for *b* of 0.01 for the investigated cation–anion pair, at least 35 environments were needed, while with five environments in the reference data set an accuracy of only 0.05 could be reached. It should be noted that for this test we used the same B^3+^—O^2−^ data set that was used in the final determination of our bond-valence parameters, where obvious outliers had already been eliminated. In practice, this removal of outliers will hardly be possible for small reference data sets, so one has to expect that the practically achievable accuracy with small reference data sets will be even worse.

The expected value of Δ*V* calculated on the training set will initially increase with the number of environments and finally converge to the internal value of the training set. This suggests that, for each cation–anion pair, there may exist a ‘true’ value of Δ*V*, but to converge reasonably well to that value requires a much larger number of environments than for *b*, which is again about 40 in this case.

**Figure 11:**
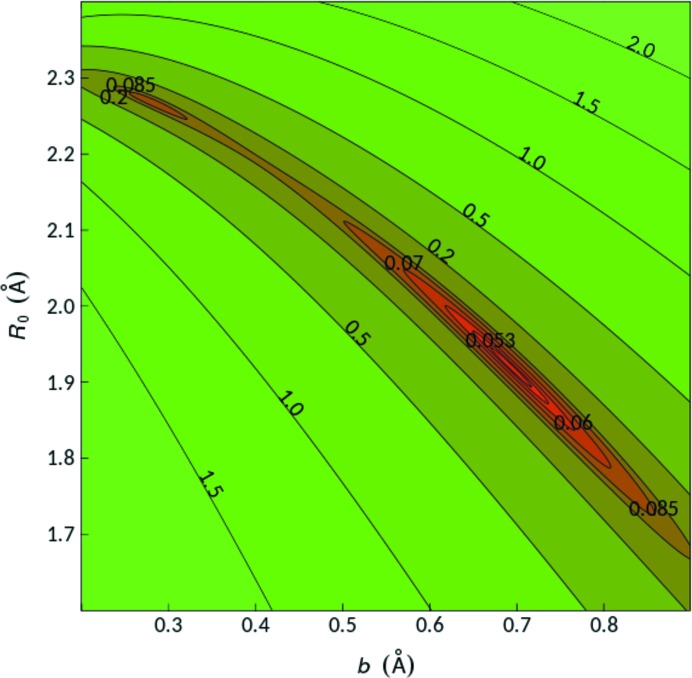
Colour-coded projection of the Δ*V* landscape as a function of *R*_0_ and *b* for our Hg^2+^—Cl^−^ reference data set, which contains *n* = 13 cation environments.

A more severe issue of refining *b* and *R*_0_ together has to do with the fundamental stability of such refinements. While some recent research suggests a nice convex landscape in the Δ*V*(*R*_0_, *b*) space (Gagné & Hawthorne, 2015 ▸), it is possible, especially when the number of environments is limited, to arrive at a Δ*V*(*R*_0_, *b*) landscape containing multiple local minima. Fig. 11 ▸ shows as an example the Δ*V*(*R*_0_, *b*) plot for our Hg^2+^—Cl^−^ reference environments set which comprises 13 Hg^2+^ environments.

This suggests that, depending on the initial choice of *R*_0_ and *b*, a minimization algorithm may fall into the wrong local minimum. It may be expected that, for a sufficient number of environments in a data set, the probability of pronounced local minima should be reduced, which further emphasizes the importance of having a sufficient number of environments for a free refinement of *b* and *R*_0_.

## 4. Summary   

In summary, we have refined two comprehensive bond softness sensitive sets of bond-valence parameters for practical use at different cut-offs, the *softBV* parameter set that comprises the weak interactions of higher coordination shells and the *softNC1* parameter set that simplifies calculations by considering only interactions in the first coordination shell. The performances of these bond-valence parameters have been compared with each other and with those of other existing parameter sets, as well as with a benchmarking parameter set that employs the traditional choice of a universally fixed value of *b* = 0.37 Å. It is found that factoring in differences in bond softness clearly improves the quality of bond-valence parameters, especially for the softer anions, while including the weak interactions of higher coordination shells improves the parameter quality only slightly.

To eliminate the bias introduced by individual decisions on the limits of the first coordination shells (which directly affects the applicability of bond-valence parameters employing the first coordination shell approach), we propose a method of systematically calculating the coordination number *N*_C_ and the cut-off distance *R*_1_ for the first coordination shell in a way that prevents the bias against extreme coordination numbers found in conventional approaches.

The profound correlation observed between our parameter set and the tried and tested Shannon crystal radii not only supports the consistency of the *N*_C_ and bond-valence parameters deduced in this work and quantifies the slight overestimation of anion sizes by Shannon, it also opens up a way of utilizing existing information on crystal radii or bond-valence parameters to generate missing information for less common cation–anion pairs.

## 5. List of symbols and abbreviations   

BV – Bond valence

BVS – Bond-valence sum

*EA* – Electron affinity

*g* – Radial distribution function

*IE* – Ionization energy

*n* – Number of cation environments in the reference data set for a cation–anion pair

*N*_C_ – Coordination number

*N*_C(Sh)_ – Coordination number from Shannon’s compilation

*N*_RCN_ – Running coordination number

*R*_csr_ – Residual value of the crystal structure refinement as listed in the ICSD

*R*_0_ – Bond-valence parameter (distance corresponding to a bond-valence value of 1 v.u.)

*R*_1_ – Radius of first coordination shell

*R*_crystal_ – Shannon crystal radius

*R*_cutoff_ – Distance up to which *M*—*X* interactions are considered to contribute to the BVS

*R*_min_ – Equilibrium distance *M*—*X* for a given coordination number

〈*R*(*M*—*X*)〉 – Expected *M*—*X* bond length

ρ(*r*) – Electron density as a function of distance *r*

ρ_BCP_ – Electron density at the bond critical point

*s*_min_ – Bond valence corresponding to *R* = *R*_min_

*V* – Bond-valence sum

*V*_id_ – Oxidation state

Δ*V* – Biased standard deviation of BVS in reference data set

η – Absolute bond hardness

σ – Absolute bond softness

χ – Mulliken electronegativity

## Supplementary Material

Comprehensive tables of bond-valence parameters, and data on the correlation between bond-valence parameters and Shannon radii.. DOI: 10.1107/S2052252517010211/yc5011sup1.pdf

## Figures and Tables

**Table 1:** Comparison of average remaining standard deviations of Δ*V* of the cations in the reference sets of cation environments for the three investigated parameter sets

Parameter set	Cut-off	Δ*V*	Δ*V* (*n* > 20)†
*softBV*	Self-consistent	0.0719	0.0807
*softNC1*	First coordination shell	0.0797	0.0891
*convBV*	First coordination shell	0.1157	0.1146

† Average standard deviation when considering only those cation–anion pairs for which the reference set comprised more than 20 cation environments.
