# Supplementary material for: Bond softness sensitive bond-valence parameters for crystal structure plausibility tests
Source: IUCrJ. 2017 Aug 2;4(Pt 5):614–25. doi: 10.1107/S2052252517010211 (PMC5619853; doi:10.1107/S2052252517010211)
Supplement: Supplementary file 1 [file m-04-00614-sup1.pdf]

IUCrJ

Volume 4 (2017)

Supporting information for article:

Bond softness sensitive bond-valence parameters for crystal structure plausibility tests

Haomin Chen and Stefan Adams

S1. Bond valence parameters of *softBV*, *softNC1* and *convBV*

Table S1 Bond valence parameters of *softBV*, *softNC1* and *convBV*.

Ions					*softBV*				*softNC1*				*convBV*			
M	V_{id}	X	V_{id}	*n*	*b*	R_0	R_{cutoff}	ΔV	N_C	R_0	R_{cutoff}	ΔV	N_C	R_0	R_{cutoff}	ΔV
Ag	1	As	-3	5	0.352	2.2683	6.50	0.069	2.80	2.2701	3.14	0.066	2.80	2.2522	3.30	0.067
Ag	1	Br	-1	6	0.470	2.0370	5.50	0.041	5.00	2.0411	3.48	0.040	5.00	2.1991	3.46	0.034
Ag	1	Cl	-1	9	0.429	1.9882	5.00	0.048	5.67	1.9931	3.36	0.054	5.67	2.0912	3.40	0.059
Ag	1	F	-1	4	0.498	1.5295	5.00	0.113	8.00	1.5653	3.32	0.162	8.00	1.8224	3.25	0.189
Ag	1	I	-1	13	0.530	2.0804	6.00	0.034	4.00	2.0929	3.59	0.030	4.00	2.3144	3.49	0.043
Ag	1	O	-2	128	0.394	1.7824	5.00	0.074	4.43	1.8171	2.97	0.083	4.66	1.8458	3.08	0.080
Ag	1	P	-3	2	0.377	2.1840	6.50	0.000	3.00	2.1859	3.15	0.003	3.00	2.1932	3.26	0.010
Ag	1	Se	-2	26	0.385	2.1741	5.00	0.081	4.38	2.1858	3.31	0.090	4.46	2.2056	3.42	0.088
Ag	1	S	-2	51	0.365	2.1101	5.00	0.062	3.63	2.1272	3.13	0.074	3.84	2.1162	3.30	0.073
Ag	1	Te	-2	11	0.420	2.3449	5.00	0.056	3.91	2.3508	3.53	0.058	3.91	2.4168	3.58	0.060
Ag	2	F	-1	16	0.509	1.5864	5.50	0.034	6.25	1.6284	2.94	0.061	6.25	1.7752	2.86	0.065
Ag	2	O	-2	2	0.501	1.7221	5.50	0.078	5.00	1.7530	2.94	0.062	5.00	1.8676	2.87	0.169
Ag	3	F	-1	4	0.452	1.7149	5.50	0.018	4.50	1.7571	2.59	0.088	4.50	1.7858	2.60	0.057
Ag	3	O	-2	4	0.444	1.8285	5.50	0.159	4.75	1.8675	2.71	0.238	4.75	1.8991	2.73	0.184
Al	3	As	-3	9	0.495	2.3664	6.50	0.141	3.89	2.3710	3.22	0.137	3.89	2.4026	3.16	0.172
Al	3	Br	-1	9	0.687	2.0404	7.00	0.046	4.00	2.0961	3.29	0.049	4.00	2.1850	2.95	0.096
Al	3	Cl	-1	33	0.646	1.8961	6.50	0.037	4.00	1.9493	3.07	0.044	4.00	2.0280	2.80	0.076
Al	3	F	-1	82	0.519	1.4197	5.50	0.061	6.00	1.4415	2.55	0.056	6.00	1.5442	2.46	0.081
Al	3	H	-1	1	0.543	1.2507	5.50	0.000	6.00	1.3326	2.49	0.000	6.00	1.4525	2.37	0.000
Al	3	I	-1	5	0.747	2.2465	7.00	0.059	4.00	2.3244	3.62	0.040	4.00	2.4313	3.20	0.066
Al	3	N	-3	9	0.511	1.6908	6.00	0.049	4.00	1.7174	2.60	0.129	4.00	1.7577	2.53	0.178
Al	3	O	-2	104	0.424	1.5990	5.00	0.101	5.33	1.6161	2.47	0.116	5.33	1.6457	2.52	0.144
Al	3	P	-3	10	0.424	2.3055	6.00	0.072	3.60	2.3067	3.00	0.070	3.60	2.3158	3.05	0.099
Al	3	Sb	-3	5	0.296	2.6398	5.00	0.124	3.40	2.6399	3.11	0.124	3.40	2.6312	3.34	0.149
Al	3	Se	-2	14	0.582	2.1739	6.50	0.063	4.00	2.2119	3.22	0.061	4.00	2.2726	3.04	0.093

Al	3	S	-2	39	0.550	2.0596	6.00	0.038	4.08	2.0945	3.06	0.046	4.08	2.1482	2.92	0.096
Al	3	Te	-2	10	0.632	2.3866	7.00	0.070	4.00	2.4310	3.53	0.071	4.00	2.5057	3.28	0.120
As	3	Cl	-1	4	0.522	2.0618	6.00	0.184	3.00	2.1083	2.86	0.203	3.00	2.1077	2.77	0.284
As	3	F	-1	5	0.406	1.6830	5.00	0.054	3.20	1.7156	2.33	0.106	3.40	1.7130	2.42	0.083
As	3	O	-2	29	0.404	1.7671	5.00	0.066	3.00	1.7894	2.37	0.109	3.00	1.7893	2.45	0.118
As	3	Se	-2	15	0.571	2.3403	6.50	0.063	3.07	2.4022	3.24	0.073	3.00	2.4032	3.07	0.175
As	3	S	-2	50	0.540	2.2084	6.00	0.076	3.20	2.2624	3.08	0.114	3.06	2.2694	2.94	0.159
As	3	Te	-2	5	0.616	2.4881	7.00	0.026	5.80	2.5319	3.83	0.069	5.80	2.6717	3.58	0.083
As	5	F	-1	6	0.503	1.6038	6.00	0.070	6.00	1.6163	2.44	0.088	6.00	1.6402	2.37	0.120
As	5	O	-2	137	0.411	1.7669	5.00	0.078	4.02	1.7793	2.28	0.102	4.04	1.7699	2.35	0.091
As	5	S	-2	6	0.534	2.2799	6.50	0.039	4.00	2.2897	2.94	0.070	4.00	2.2530	2.83	0.101
Au	1	O	-2	4	0.441	1.7182	5.50	0.043	2.00	1.7263	2.67	0.041	2.00	1.7755	2.69	0.048
Au	1	Se	-2	7	0.346	2.1761	4.00	0.047	2.00	2.1836	2.92	0.038	2.00	2.1670	3.09	0.036
Au	1	S	-2	13	0.342	2.0640	4.50	0.034	2.00	2.0685	2.80	0.030	2.00	2.0491	2.97	0.028
Au	3	Br	-1	7	0.461	2.2791	5.50	0.028	4.00	2.2942	3.09	0.037	4.00	2.3203	3.09	0.046
Au	3	Cl	-1	17	0.421	2.1452	5.50	0.029	4.00	2.1591	2.89	0.052	4.00	2.1736	2.94	0.059
Au	3	F	-1	36	0.507	1.6970	5.50	0.068	4.00	1.7665	2.65	0.072	4.00	1.8056	2.58	0.097
Au	3	O	-2	11	0.498	1.8176	5.50	0.096	4.00	1.8577	2.72	0.105	4.00	1.8944	2.66	0.140
B	3	As	-3	3	0.566	2.0965	6.50	0.037	2.00	2.1001	2.69	0.032	2.00	2.0206	2.53	0.049
B	3	F	-1	19	0.542	1.2004	5.00	0.050	4.00	1.2282	2.17	0.061	4.00	1.2774	2.05	0.092
B	3	H	-1	1	0.565	0.9866	5.50	0.000	4.00	1.0503	2.03	0.000	4.00	1.1057	1.88	0.000
B	3	N	-3	11	0.301	1.4568	4.00	0.086	2.55	1.4587	1.84	0.090	2.55	1.4720	2.07	0.145
B	3	O	-2	315	0.385	1.3578	4.50	0.057	3.41	1.3681	1.97	0.061	3.41	1.3699	2.08	0.061
B	3	P	-3	8	0.284	1.8845	5.00	0.023	2.50	1.8849	2.24	0.024	2.50	1.9040	2.50	0.214
B	3	S	-2	13	0.573	1.7819	6.00	0.074	3.00	1.8104	2.64	0.061	3.00	1.8101	2.47	0.095
Ba	2	As	-3	10	0.674	2.6084	8.00	0.089	6.20	2.6411	4.38	0.097	6.00	2.9775	4.05	0.138
Ba	2	Br	-1	7	0.595	2.4688	7.00	0.042	8.57	2.4892	4.22	0.045	8.57	2.8083	4.01	0.069
Ba	2	Cl	-1	5	0.519	2.4104	6.50	0.021	9.60	2.4231	3.99	0.035	9.00	2.6504	3.87	0.058
Ba	2	F	-1	71	0.428	2.0648	6.00	0.093	11.31	2.0793	3.44	0.097	11.34	2.1745	3.48	0.105
Ba	2	H	-1	6	0.641	1.6245	6.00	0.073	12.33	1.6765	3.77	0.052	12.17	2.1596	3.49	0.103

Ba	2	I	-1	10	0.655	2.6138	7.50	0.103	8.60	2.6424	4.55	0.107	8.50	3.0457	4.24	0.230
Ba	2	N	-3	34	0.525	2.2680	6.50	0.127	6.97	2.2976	3.71	0.139	6.85	2.4767	3.60	0.171
Ba	2	O	-2	103	0.437	2.1600	6.00	0.158	9.87	2.1758	3.51	0.174	9.95	2.2762	3.53	0.176
Ba	2	P	-3	18	0.644	2.5673	7.50	0.129	6.22	2.5975	4.26	0.125	6.22	2.9046	3.99	0.175
Ba	2	Sb	-3	8	0.749	2.6859	8.50	0.120	6.00	2.7317	4.64	0.115	6.00	3.1441	4.21	0.226
Ba	2	Se	-2	17	0.604	2.5198	7.00	0.080	8.29	2.5427	4.28	0.092	8.06	2.8663	4.04	0.130
Ba	2	S	-2	37	0.573	2.4424	7.00	0.132	8.97	2.4610	4.15	0.135	8.84	2.7553	3.97	0.200
Ba	2	Te	-2	5	0.649	2.6881	7.00	0.033	7.40	2.7112	4.50	0.049	7.40	3.0637	4.21	0.186
Be	2	As	-3	3	0.379	2.0569	8.00	0.027	3.00	2.0586	2.76	0.028	3.00	2.0619	2.87	0.034
Be	2	Cl	-1	2	0.659	1.5273	6.50	0.083	4.00	1.5654	2.98	0.016	4.00	1.7655	2.68	0.027
Be	2	F	-1	34	0.532	1.1499	5.50	0.058	3.97	1.1802	2.31	0.076	3.97	1.2895	2.21	0.096
Be	2	N	-3	5	0.629	1.3123	6.00	0.035	3.00	1.3529	2.52	0.084	3.00	1.4571	2.27	0.140
Be	2	O	-2	37	0.541	1.2090	5.50	0.049	4.00	1.2608	2.42	0.053	4.00	1.3785	2.30	0.077
Be	2	Sb	-3	2	0.479	2.1921	6.00	0.000	3.50	2.2015	3.16	0.014	3.50	2.2605	3.13	0.101
Bi	3	Br	-1	3	0.545	2.4671	7.00	0.019	6.00	2.4856	3.65	0.123	6.00	2.5950	3.51	0.050
Bi	3	Cl	-1	6	0.505	2.3411	6.00	0.058	6.33	2.3486	3.46	0.059	6.00	2.4400	3.36	0.084
Bi	3	F	-1	10	0.423	1.9305	5.50	0.101	7.10	1.9345	2.91	0.107	7.10	1.9774	2.96	0.145
Bi	3	I	-1	8	0.605	2.6399	7.50	0.035	6.00	2.6613	3.96	0.059	6.00	2.8208	3.74	0.134
Bi	3	O	-2	86	0.414	2.0368	5.50	0.105	6.05	2.0586	2.95	0.146	6.09	2.0824	3.01	0.177
Bi	3	Se	-2	19	0.554	2.5455	7.00	0.054	6.11	2.5630	3.76	0.048	6.11	2.6826	3.61	0.077
Bi	3	S	-2	12	0.523	2.4182	6.50	0.030	7.25	2.4411	3.66	0.060	7.00	2.5526	3.53	0.085
Bi	3	Te	-2	2	0.599	2.8230	7.00	0.032	4.50	2.8569	3.97	0.128	4.50	2.9329	3.75	0.433
Bi	5	F	-1	3	0.448	1.8698	5.50	0.088	6.00	1.8815	2.61	0.088	6.00	1.8948	2.63	0.108
Bi	5	O	-2	6	0.371	2.0450	5.00	0.109	6.00	2.0475	2.65	0.089	6.00	2.0476	2.78	0.090
Bk	3	O	-2	3	0.430	2.0307	6.00	0.020	6.67	2.0448	3.01	0.033	6.67	2.0891	3.05	0.018
Br	5	F	-1	1	0.370	1.7848	7.00	0.000	6.00	1.7865	2.39	0.000	6.00	1.7865	2.52	0.000
Br	5	O	-2	25	0.444	1.8509	5.50	0.054	3.00	1.8796	2.30	0.058	3.00	1.8418	2.32	0.070
Br	7	O	-2	7	0.423	1.8368	5.50	0.031	4.00	1.8426	2.22	0.051	4.00	1.8129	2.27	0.059
C	2	F	-1	3	0.407	1.1591	6.00	0.037	3.00	1.1842	1.94	0.030	3.00	1.1992	2.01	0.033
C	2	O	-2	386	0.415	1.4137	5.00	0.070	1.00	1.4250	1.74	0.070	1.00	1.3938	1.80	0.079

C	4	F	-1	3	0.547	1.3725	6.00	0.052	3.00	1.4458	2.08	0.085	3.00	1.3947	1.95	0.126
C	4	N	-3	12	0.529	1.5579	6.00	0.079	2.00	1.5885	1.99	0.100	2.00	1.4774	1.88	0.144
C	4	O	-2	73	0.447	1.3983	5.00	0.044	3.00	1.4113	1.93	0.038	3.00	1.3891	1.95	0.046
C	4	S	-2	4	0.579	1.8435	6.00	0.068	3.00	1.8739	2.55	0.035	3.00	1.8130	2.37	0.059
Ca	2	As	-3	13	0.713	2.2057	8.00	0.039	6.00	2.2648	4.08	0.034	5.85	2.6352	3.70	0.059
Ca	2	Cl	-1	2	0.594	2.0626	6.50	0.000	6.00	2.0914	3.60	0.000	6.00	2.3370	3.41	0.000
Ca	2	F	-1	19	0.467	1.7104	5.50	0.075	6.89	1.7369	2.99	0.104	6.89	1.8551	2.98	0.112
Ca	2	H	-1	5	0.490	1.6066	5.50	0.128	10.20	1.6357	3.14	0.137	10.20	1.8271	3.09	0.142
Ca	2	N	-3	12	0.564	1.9698	6.50	0.108	5.17	2.0128	3.36	0.112	5.17	2.1910	3.21	0.095
Ca	2	O	-2	125	0.476	1.7952	5.50	0.115	7.55	1.8279	3.15	0.112	7.54	1.9625	3.12	0.131
Ca	2	P	-3	8	0.684	2.1950	7.50	0.118	6.00	2.2442	3.99	0.110	5.88	2.5814	3.64	0.183
Ca	2	Sb	-3	6	0.788	2.3144	8.00	0.073	6.00	2.3827	4.39	0.067	5.83	2.8359	3.89	0.141
Ca	2	Se	-2	4	0.643	2.2028	7.00	0.051	7.00	2.2389	3.97	0.058	7.00	2.5764	3.70	0.187
Ca	2	S	-2	20	0.612	2.1115	7.00	0.095	7.20	2.1527	3.82	0.087	7.20	2.4570	3.59	0.178
Cd	2	As	-3	9	0.400	2.4246	7.50	0.221	3.78	2.4285	3.26	0.212	3.78	2.4469	3.35	0.218
Cd	2	Br	-1	11	0.553	2.1691	6.00	0.062	5.27	2.1781	3.51	0.035	5.27	2.3499	3.37	0.194
Cd	2	Cl	-1	8	0.512	2.0695	6.00	0.048	6.00	2.0779	3.38	0.048	6.00	2.2331	3.30	0.068
Cd	2	F	-1	16	0.416	1.7395	5.50	0.036	6.81	1.7532	2.86	0.046	6.81	1.8085	2.92	0.061
Cd	2	I	-1	9	0.613	2.3244	7.00	0.042	5.44	2.3371	3.84	0.034	5.33	2.5635	3.59	0.173
Cd	2	O	-2	90	0.407	1.8393	5.50	0.093	6.22	1.8609	2.91	0.101	6.33	1.9003	2.99	0.107
Cd	2	P	-3	9	0.370	2.3533	7.50	0.277	3.56	2.3562	3.10	0.267	3.56	2.3562	3.23	0.267
Cd	2	Se	-2	7	0.561	2.1621	6.50	0.109	4.00	2.2156	3.42	0.081	4.00	2.3477	3.27	0.123
Cd	2	S	-2	23	0.531	2.1279	5.50	0.041	4.43	2.1688	3.36	0.057	4.30	2.2928	3.24	0.080
Cd	2	Te	-2	5	0.607	2.3668	7.00	0.142	4.00	2.4000	3.70	0.117	4.00	2.5628	3.48	0.188
Ce	3	F	-1	4	0.458	1.9266	5.50	0.064	9.75	1.9387	3.14	0.058	9.75	2.0375	3.14	0.056
Ce	3	O	-2	34	0.449	2.0312	5.50	0.081	9.06	2.0485	3.19	0.093	9.06	2.1315	3.20	0.130
Ce	3	Se	-2	1	0.520	2.5266	7.00	0.000	9.00	2.5422	3.87	0.000	9.00	2.7059	3.78	0.000
Ce	3	S	-2	3	0.489	2.5040	6.00	0.120	7.33	2.5185	3.66	0.084	7.33	2.6178	3.61	0.019
Ce	4	O	-2	15	0.443	2.0253	5.50	0.059	8.00	2.0379	2.99	0.080	8.00	2.0863	3.01	0.143
Cl	5	O	-2	11	0.445	1.6955	5.50	0.040	3.00	1.7109	2.13	0.059	3.00	1.6726	2.15	0.071

Cl	7	O	-2	20	0.443	1.6795	5.00	0.084	4.00	1.6865	2.08	0.095	4.00	1.6453	2.10	0.107
Co	1	O	-2	5	0.621	1.2950	5.50	0.014	2.00	1.3186	2.65	0.010	2.00	1.4925	2.41	0.017
Co	2	Cl	-1	16	0.468	1.9314	5.50	0.048	4.63	1.9360	3.01	0.054	4.63	2.0155	2.99	0.143
Co	2	F	-1	30	0.460	1.5144	5.50	0.095	6.00	1.5291	2.70	0.078	6.00	1.6277	2.70	0.097
Co	2	I	-1	2	0.569	2.1984	6.00	0.002	4.00	2.2038	3.42	0.004	4.00	2.3417	3.26	0.005
Co	2	O	-2	166	0.451	1.5977	5.50	0.112	5.51	1.6238	2.73	0.110	5.51	1.7034	2.74	0.109
Co	2	P	-3	4	0.734	1.6236	7.50	0.043	4.00	1.7444	3.32	0.054	4.00	1.9960	2.92	0.104
Co	2	Se	-2	5	0.518	2.1095	6.00	0.034	4.00	2.1135	3.22	0.036	4.00	2.2158	3.14	0.049
Co	2	S	-2	8	0.487	2.0005	5.50	0.057	4.00	2.0052	3.05	0.049	4.00	2.0862	3.01	0.065
Co	3	F	-1	5	0.443	1.5635	5.50	0.068	6.00	1.5732	2.52	0.057	6.00	1.6238	2.54	0.068
Co	3	O	-2	12	0.434	1.5946	5.50	0.119	6.00	1.6090	2.54	0.118	6.00	1.6532	2.57	0.136
Co	4	O	-2	5	0.441	1.7944	5.50	0.096	4.00	1.7983	2.44	0.101	4.00	1.7981	2.46	0.119
Cr	2	Br	-1	2	0.480	2.1268	6.00	0.038	6.00	2.1325	3.35	0.023	6.00	2.2467	3.32	0.048
Cr	2	Cl	-1	6	0.439	2.0150	5.50	0.031	6.00	2.0194	3.14	0.027	6.00	2.0892	3.16	0.038
Cr	2	F	-1	11	0.489	1.5410	5.50	0.048	5.64	1.5759	2.79	0.126	5.64	1.6903	2.74	0.092
Cr	2	I	-1	4	0.540	2.2840	6.50	0.011	6.00	2.2884	3.66	0.012	6.00	2.4680	3.54	0.028
Cr	2	O	-2	15	0.480	1.5936	5.50	0.072	5.60	1.6328	2.82	0.072	5.60	1.7381	2.78	0.072
Cr	2	S	-2	3	0.458	1.9093	5.50	0.149	6.00	1.9207	3.09	0.139	6.00	2.0163	3.09	0.167
Cr	2	Te	-2	4	0.534	2.1826	6.50	0.031	6.00	2.1972	3.56	0.024	6.00	2.3772	3.45	0.035
Cr	3	Br	-1	5	0.530	2.1316	6.50	0.034	6.00	2.1499	3.28	0.076	6.00	2.2602	3.18	0.118
Cr	3	Cl	-1	5	0.489	2.0051	5.50	0.048	6.00	2.0120	3.06	0.038	6.00	2.0940	3.01	0.054
Cr	3	F	-1	50	0.438	1.5897	5.50	0.043	6.00	1.5986	2.54	0.036	6.00	1.6456	2.56	0.043
Cr	3	O	-2	55	0.430	1.6620	5.50	0.071	6.00	1.6777	2.60	0.067	6.00	1.7190	2.64	0.077
Cr	3	Se	-2	13	0.539	2.1465	6.00	0.057	6.00	2.1675	3.32	0.047	6.00	2.2839	3.20	0.071
Cr	3	S	-2	36	0.508	2.0440	5.50	0.038	6.00	2.0640	3.15	0.036	6.00	2.1589	3.08	0.051
Cr	3	Te	-2	3	0.584	2.3024	6.50	0.030	6.00	2.3269	3.58	0.037	6.00	2.4752	3.39	0.058
Cr	4	F	-1	7	0.418	1.6490	5.00	0.149	6.00	1.6569	2.43	0.150	6.00	1.6758	2.49	0.173
Cr	4	O	-2	7	0.409	1.7609	5.50	0.076	5.43	1.7675	2.48	0.064	5.43	1.7785	2.55	0.136
Cr	5	O	-2	3	0.402	1.7678	5.50	0.142	4.00	1.7764	2.27	0.148	4.00	1.7690	2.35	0.164
Cr	6	N	-3	2	0.605	1.9686	6.50	0.027	4.00	2.0077	2.64	0.074	4.00	1.9123	2.43	0.122

Cr	6	O	-2	99	0.476	1.8247	5.50	0.088	4.00	1.8414	2.34	0.103	4.00	1.7974	2.31	0.133
Cr	6	Te	-2	2	0.729	2.6086	8.00	0.011	6.00	2.7287	3.78	0.069	6.00	2.7285	3.39	0.134
Cs	1	As	-3	24	0.619	2.7643	7.50	0.080	5.29	2.7812	4.71	0.079	5.08	3.1787	4.44	0.137
Cs	1	Br	-1	39	0.541	2.5082	7.00	0.063	10.31	2.5152	4.56	0.062	10.28	2.9025	4.43	0.111
Cs	1	Cl	-1	78	0.500	2.4604	6.50	0.095	10.33	2.4669	4.36	0.094	10.29	2.7635	4.29	0.111
Cs	1	F	-1	51	0.428	2.1532	6.00	0.056	11.55	2.1666	3.83	0.054	11.55	2.3040	3.87	0.056
Cs	1	H	-1	5	0.404	2.2466	6.00	0.084	8.80	2.2516	3.71	0.085	8.80	2.3250	3.79	0.088
Cs	1	I	-1	31	0.601	2.7119	8.00	0.041	10.48	2.7241	5.01	0.047	10.42	3.2552	4.79	0.105
Cs	1	N	-3	4	0.471	2.4310	6.00	0.036	11.00	2.4436	4.25	0.021	11.00	2.6775	4.23	0.025
Cs	1	Nx[#1]	-3	19	0.471	2.5621	6.00	0.099	10.89	2.5656	4.37	0.092	10.89	2.7956	4.34	0.182
Cs	1	O	-2	38	0.419	2.2590	6.50	0.078	11.50	2.2758	3.91	0.080	11.63	2.3883	3.96	0.080
Cs	1	P	-3	18	0.590	2.7298	7.00	0.032	4.94	2.7449	4.54	0.036	4.89	3.0818	4.33	0.079
Cs	1	Sb	-3	7	0.694	2.9184	8.00	0.047	5.14	2.9446	5.09	0.052	5.14	3.4646	4.73	0.097
Cs	1	Se	-2	37	0.550	2.6486	7.00	0.079	7.27	2.6661	4.56	0.081	7.16	3.0110	4.40	0.102
Cs	1	S	-2	41	0.519	2.5228	6.50	0.064	7.56	2.5396	4.34	0.061	7.41	2.8328	4.24	0.095
Cs	1	Te	-2	6	0.595	2.7823	7.50	0.077	7.50	2.7920	4.85	0.080	7.00	3.2257	4.61	0.099
Cu	1	Br	-1	10	0.442	1.8681	5.50	0.045	3.80	1.8773	3.11	0.044	3.80	1.9724	3.13	0.047
Cu	1	Cl	-1	14	0.402	1.7982	5.00	0.028	3.86	1.8066	2.93	0.028	3.86	1.8490	3.01	0.029
Cu	1	I	-1	13	0.503	1.9307	6.00	0.044	3.92	1.9441	3.36	0.044	3.85	2.1246	3.29	0.049
Cu	1	O	-2	82	0.341	1.5873	5.00	0.073	2.48	1.6084	2.41	0.087	2.61	1.5768	2.62	0.078
Cu	1	P	-3	13	0.402	1.9275	6.50	0.077	3.08	1.9377	2.97	0.083	3.15	1.9706	3.06	0.077
Cu	1	Se	-2	19	0.451	1.8956	5.50	0.068	3.74	1.9094	3.16	0.071	3.74	2.0126	3.16	0.074
Cu	1	S	-2	104	0.420	1.8074	5.00	0.089	3.35	1.8225	2.94	0.100	3.36	1.8819	2.99	0.096
Cu	1	Te	-2	15	0.497	1.8898	6.00	0.039	4.00	1.9020	3.31	0.044	4.00	2.0779	3.25	0.059
Cu	2	Cl	-1	13	0.470	1.9018	5.50	0.054	5.62	1.9069	3.07	0.061	5.62	1.9989	3.04	0.090
Cu	2	F	-1	74	0.457	1.4953	5.50	0.076	5.84	1.5124	2.66	0.092	5.84	1.6006	2.66	0.093
Cu	2	O	-2	310	0.449	1.5742	5.00	0.067	5.09	1.6015	2.67	0.089	5.10	1.6703	2.68	0.079
Cu	2	Se	-2	4	0.519	2.0289	6.00	0.040	4.00	2.0577	3.17	0.031	4.00	2.1609	3.08	0.044
Cu	2	S	-2	25	0.488	1.9500	6.00	0.090	3.88	1.9783	3.01	0.083	3.88	2.0556	2.96	0.107
Cu	3	F	-1	3	0.437	1.5427	5.50	0.210	6.00	1.5463	2.48	0.229	6.00	1.5925	2.51	0.269

Cu	3	O	-2	11	0.427	1.7096	5.00	0.063	4.00	1.7177	2.46	0.057	4.00	1.7340	2.50	0.066
Dy	3	Cl	-1	6	0.493	2.2881	6.00	0.046	7.00	2.2941	3.43	0.044	7.00	2.3969	3.37	0.107
Dy	3	O	-2	29	0.426	1.9603	5.50	0.095	7.62	1.9732	2.99	0.096	7.62	2.0238	3.03	0.113
Dy	3	S	-2	7	0.512	2.3412	6.00	0.094	7.43	2.3552	3.56	0.088	7.43	2.4808	3.48	0.166
Er	3	Cl	-1	3	0.507	2.2598	6.00	0.017	6.33	2.2690	3.38	0.021	6.33	2.3699	3.31	0.097
Er	3	F	-1	16	0.421	1.8466	5.50	0.102	8.13	1.8550	2.88	0.107	8.13	1.9046	2.94	0.106
Er	3	O	-2	37	0.412	1.9561	5.50	0.082	7.08	1.9685	2.92	0.081	7.08	2.0034	2.98	0.087
Er	3	Se	-2	6	0.556	2.4342	7.00	0.056	6.00	2.4519	3.64	0.047	6.00	2.5801	3.50	0.070
Er	3	S	-2	16	0.525	2.3237	6.00	0.089	6.38	2.3412	3.50	0.091	6.38	2.4556	3.40	0.139
Eu	2	Br	-1	3	0.465	2.5001	6.00	0.064	7.33	2.5099	3.79	0.065	7.33	2.6311	3.77	0.094
Eu	2	Cl	-1	2	0.425	2.4145	5.50	0.003	9.00	2.4186	3.67	0.003	9.00	2.4968	3.72	0.003
Eu	2	F	-1	2	0.503	1.8142	5.50	0.069	7.00	1.8249	3.18	0.049	7.00	1.9902	3.12	0.037
Eu	2	I	-1	2	0.525	2.6629	6.50	0.010	6.50	2.6736	4.05	0.011	6.50	2.8543	3.95	0.080
Eu	2	O	-2	9	0.494	1.8916	6.00	0.077	10.11	1.9154	3.43	0.099	10.00	2.1079	3.37	0.136
Eu	2	S	-2	19	0.443	2.4717	6.00	0.056	7.63	2.4813	3.72	0.059	7.63	2.5768	3.74	0.061
Eu	3	Cl	-1	5	0.485	2.3200	6.00	0.009	8.20	2.3308	3.52	0.017	8.20	2.4450	3.48	0.092
Eu	3	F	-1	3	0.443	1.8776	5.50	0.018	8.00	1.8893	2.96	0.017	8.00	1.9599	2.99	0.020
Eu	3	O	-2	35	0.434	2.0047	5.50	0.083	7.63	2.0189	3.05	0.082	7.69	2.0751	3.09	0.092
Eu	3	S	-2	6	0.503	2.3918	6.00	0.154	7.00	2.4023	3.56	0.157	7.00	2.5135	3.49	0.121
Fe	2	Br	-1	5	0.479	2.0952	5.50	0.158	6.00	2.1009	3.32	0.166	6.00	2.2162	3.29	0.215
Fe	2	Cl	-1	5	0.439	2.0000	5.00	0.036	6.00	2.0054	3.12	0.035	6.00	2.0802	3.15	0.039
Fe	2	F	-1	26	0.489	1.5077	5.50	0.096	6.00	1.5282	2.77	0.078	6.00	1.6561	2.73	0.128
Fe	2	H	-1	6	0.466	1.0507	5.50	0.092	6.00	1.0676	2.25	0.078	6.00	1.1730	2.24	0.098
Fe	2	I	-1	2	0.540	2.2579	6.00	0.019	5.00	2.2655	3.54	0.029	5.00	2.4149	3.42	0.225
Fe	2	N	-3	6	0.367	1.7473	5.50	0.043	4.00	1.7514	2.54	0.047	4.00	1.7495	2.67	0.045
Fe	2	O	-2	113	0.480	1.5791	5.50	0.097	5.82	1.6218	2.83	0.105	5.76	1.7347	2.79	0.127
Fe	2	P	-3	4	0.680	1.7103	6.50	0.060	4.00	1.7898	3.25	0.063	4.00	2.0043	2.92	0.113
Fe	2	S	-2	30	0.457	2.0111	5.50	0.129	5.33	2.0236	3.13	0.125	5.33	2.1063	3.13	0.139
Fe	3	Br	-1	4	0.578	2.1363	6.50	0.107	4.00	2.1676	3.17	0.095	4.00	2.2270	3.00	0.148
Fe	3	Cl	-1	21	0.538	1.9826	6.00	0.110	5.43	2.0042	3.10	0.115	5.43	2.1000	2.98	0.203

Fe	3	F	-1	59	0.411	1.6367	5.00	0.060	5.98	1.6424	2.52	0.063	5.98	1.6703	2.59	0.066
Fe	3	I	-1	2	0.523	2.3766	6.00	0.000	4.00	2.3842	3.29	0.000	4.00	2.4281	3.20	0.000
Fe	3	N	-3	3	0.606	1.7248	6.00	0.063	3.33	1.7603	2.70	0.111	3.33	1.7800	2.48	0.394
Fe	3	O	-2	165	0.420	1.7084	5.00	0.100	5.73	1.7211	2.60	0.112	5.73	1.7525	2.66	0.104
Fe	4	O	-2	2	0.410	1.7656	5.50	0.025	6.00	1.7695	2.53	0.021	6.00	1.7857	2.60	0.024
Ga	1	Cl	-1	3	0.440	2.3204	6.00	0.072	9.00	2.3288	3.93	0.072	9.00	2.4745	3.95	0.087
Ga	1	I	-1	2	0.541	2.3877	7.00	0.022	8.00	2.4014	4.31	0.024	8.00	2.7428	4.18	0.025
Ga	3	As	-3	6	0.466	2.4181	6.50	0.044	3.33	2.4205	3.14	0.049	3.33	2.4292	3.13	0.116
Ga	3	Br	-1	4	0.619	2.1135	6.50	0.026	4.00	2.1461	3.22	0.026	4.00	2.2175	2.99	0.045
Ga	3	Cl	-1	13	0.578	1.9779	6.50	0.034	4.00	2.0128	3.02	0.040	4.00	2.0705	2.84	0.049
Ga	3	F	-1	13	0.451	1.5604	5.50	0.035	6.00	1.5706	2.54	0.036	6.00	1.6261	2.55	0.049
Ga	3	H	-1	3	0.474	1.4078	6.00	0.257	4.00	1.4236	2.25	0.283	4.00	1.4528	2.22	0.360
Ga	3	N	-3	5	0.506	1.7620	6.00	0.034	4.00	1.7820	2.66	0.092	4.00	1.8207	2.59	0.128
Ga	3	O	-2	84	0.373	1.7161	5.00	0.109	4.90	1.7236	2.45	0.109	4.90	1.7250	2.57	0.110
Ga	3	P	-3	7	0.433	2.3184	6.00	0.097	3.43	2.3192	3.00	0.095	3.43	2.3264	3.04	0.124
Ga	3	Sb	-3	2	0.669	2.6418	7.00	0.017	3.00	2.6449	3.61	0.024	3.00	2.6446	3.31	0.046
Ga	3	Se	-2	30	0.513	2.2472	6.50	0.113	4.00	2.2662	3.16	0.102	4.00	2.3065	3.08	0.142
Ga	3	S	-2	52	0.483	2.1246	6.00	0.079	4.00	2.1427	2.98	0.073	4.00	2.1747	2.94	0.097
Ga	3	Te	-2	11	0.558	2.4449	7.00	0.100	4.00	2.4749	3.44	0.096	4.00	2.5284	3.30	0.146
Gd	3	Cl	-1	4	0.533	2.2713	6.00	0.068	7.75	2.2854	3.56	0.063	7.75	2.4381	3.45	0.063
Gd	3	F	-1	3	0.406	1.9072	5.50	0.010	8.33	1.9141	2.92	0.016	8.33	1.9502	2.99	0.016
Gd	3	O	-2	77	0.415	1.9965	5.50	0.120	7.77	2.0109	3.01	0.138	7.91	2.0505	3.07	0.139
Gd	3	S	-2	5	0.552	2.3703	6.00	0.185	7.20	2.3845	3.67	0.171	7.20	2.5404	3.53	0.094
Ge	2	As	-3	3	0.458	2.3808	6.50	0.028	2.00	2.4137	3.08	0.045	2.00	2.4137	3.08	0.055
Ge	2	Br	-1	4	0.531	2.2006	6.00	0.105	6.00	2.2039	3.56	0.113	5.50	2.3466	3.38	0.157
Ge	2	Cl	-1	3	0.490	2.0749	5.50	0.072	5.00	2.0857	3.24	0.104	5.00	2.1706	3.17	0.116
Ge	2	I	-1	3	0.591	2.2913	7.00	0.147	6.00	2.3074	3.81	0.136	6.00	2.5378	3.61	0.218
Ge	2	N	-3	2	0.461	1.8612	6.00	0.054	2.00	1.8708	2.54	0.031	2.00	1.8708	2.53	0.038
Ge	2	P	-3	3	0.580	2.2278	6.50	0.045	2.00	2.3116	3.15	0.072	2.00	2.3114	2.97	0.113
Ge	2	Te	-2	9	0.585	2.2694	7.00	0.025	6.00	2.2931	3.78	0.025	6.00	2.5270	3.60	0.028

Ge	4	As	-3	7	0.465	2.4695	6.00	0.077	3.86	2.4709	3.13	0.077	3.86	2.4668	3.12	0.124
Ge	4	Cl	-1	2	0.610	2.0762	6.00	0.104	5.00	2.0981	3.12	0.017	5.00	2.1432	2.89	0.551
Ge	4	F	-1	20	0.484	1.5786	5.50	0.099	6.00	1.5916	2.49	0.087	6.00	1.6377	2.45	0.114
Ge	4	I	-1	2	0.711	2.4339	7.50	0.017	4.00	2.5000	3.53	0.007	4.00	2.5000	3.16	0.013
Ge	4	N	-3	3	0.469	1.8476	6.00	0.140	4.00	1.8722	2.55	0.156	4.00	1.8719	2.53	0.199
Ge	4	O	-2	167	0.396	1.7394	5.00	0.101	4.29	1.7504	2.35	0.114	4.29	1.7517	2.44	0.118
Ge	4	P	-3	8	0.552	2.3138	6.50	0.149	4.00	2.3333	3.13	0.141	4.00	2.3331	3.00	0.206
Ge	4	Se	-2	19	0.545	2.3326	6.00	0.101	4.00	2.3509	3.14	0.101	4.00	2.3503	3.01	0.149
Ge	4	S	-2	40	0.514	2.1918	6.00	0.073	4.00	2.2120	2.96	0.081	4.00	2.2116	2.87	0.112
Ge	4	Te	-2	3	0.593	2.5746	7.00	0.129	3.67	2.6514	3.46	0.296	3.67	2.6313	3.26	0.167
H	1	Br	-1	5	0.725	1.0705	6.00	0.024	1.80	1.2286	2.70	0.045	1.00	1.3146	1.98	0.092
H	1	Cl	-1	3	0.685	1.0104	5.50	0.046	1.67	1.1418	2.61	0.046	1.00	1.2316	1.89	0.138
H	1	F	-1	5	0.558	0.7078	4.50	0.021	1.80	0.7790	1.91	0.120	1.80	0.8872	1.77	0.058
H	1	I	-1	2	0.786	1.1778	6.50	0.004	1.00	1.4483	2.59	0.022	1.00	1.4481	2.11	0.047
H	1	O	-2	244	0.457	0.8704	4.00	0.033	1.40	0.9337	1.75	0.074	1.37	0.9520	1.73	0.059
H	1	S	-2	5	0.591	1.1916	5.50	0.039	1.00	1.2917	2.15	0.062	1.00	1.2910	1.95	0.099
Hf	4	Cl	-1	5	0.483	2.2444	6.50	0.023	6.00	2.2516	3.15	0.039	6.00	2.2963	3.11	0.055
Hf	4	F	-1	4	0.469	1.7752	5.50	0.011	7.25	1.7846	2.74	0.009	7.25	1.8424	2.73	0.066
Hf	4	I	-1	4	0.584	2.5768	7.00	0.044	6.00	2.5922	3.67	0.004	6.00	2.6725	3.49	0.040
Hf	4	O	-2	19	0.478	1.8336	6.00	0.081	7.11	1.8560	2.82	0.116	7.11	1.9159	2.79	0.187
Hf	4	Se	-2	4	0.645	2.3789	7.50	0.071	6.00	2.4176	3.61	0.060	6.00	2.5285	3.34	0.104
Hf	4	S	-2	6	0.615	2.2355	7.00	0.151	6.00	2.2615	3.40	0.217	6.00	2.3600	3.17	0.358
Hg	1	Cl	-1	3	0.455	2.1734	5.50	0.103	5.00	2.1974	3.59	0.127	5.00	2.2934	3.55	0.156
Hg	1	F	-1	3	0.473	1.7651	5.50	0.045	5.00	1.7882	3.23	0.050	5.00	1.9356	3.19	0.079
Hg	1	O	-2	14	0.465	1.8128	5.50	0.051	4.43	1.8609	3.26	0.044	4.14	1.9870	3.18	0.080
Hg	2	Br	-1	7	0.495	2.2544	6.00	0.061	5.00	2.2652	3.44	0.082	5.00	2.3547	3.36	0.143
Hg	2	Cl	-1	13	0.455	2.1514	5.50	0.093	5.23	2.1649	3.27	0.186	5.08	2.2247	3.23	0.150
Hg	2	F	-1	7	0.473	1.7338	5.50	0.081	6.29	1.7395	2.97	0.080	6.29	1.8564	2.94	0.121
Hg	2	I	-1	10	0.555	2.3816	6.00	0.029	4.00	2.4001	3.59	0.047	4.00	2.5281	3.45	0.071
Hg	2	O	-2	29	0.465	1.8128	5.50	0.094	6.48	1.8514	3.07	0.113	6.34	1.9437	3.03	0.118

Hg	2	Se	-2	5	0.504	2.2518	6.00	0.036	4.00	2.2745	3.35	0.044	4.00	2.3674	3.29	0.061
Hg	2	S	-2	18	0.473	2.2050	5.50	0.070	4.39	2.2344	3.29	0.086	4.28	2.3049	3.25	0.097
Hg	2	Te	-2	3	0.549	2.3792	7.00	0.071	4.00	2.3976	3.57	0.057	4.00	2.5215	3.44	0.085
Ho	3	F	-1	9	0.424	1.8448	5.50	0.060	8.33	1.8550	2.90	0.062	8.33	1.9091	2.95	0.068
Ho	3	O	-2	46	0.415	1.9710	5.50	0.111	7.37	1.9813	2.95	0.114	7.39	2.0199	3.02	0.126
Ho	3	S	-2	8	0.522	2.3380	6.00	0.068	6.88	2.3542	3.54	0.068	6.88	2.4759	3.45	0.151
Hx[#2]	1	F	-1	6	0.558	0.6665	4.50	0.050	2.00	0.7239	1.92	0.057	2.00	0.8325	1.75	0.137
Hx	1	S	-2	12	0.591	1.0978	5.50	0.061	1.83	1.1676	2.67	0.093	1.00	1.2623	1.93	0.157
I	5	F	-1	13	0.370	1.8557	7.00	0.146	4.92	1.8747	2.40	0.203	5.69	1.8647	2.58	0.131
I	5	O	-2	67	0.424	1.9777	6.00	0.108	3.07	2.0255	2.43	0.151	3.09	1.9983	2.48	0.174
I	7	O	-2	5	0.419	1.9227	5.50	0.116	5.80	1.9279	2.46	0.144	5.80	1.9179	2.51	0.221
In	1	Br	-1	10	0.454	2.4666	6.00	0.037	8.40	2.4747	4.10	0.030	8.40	2.6436	4.09	0.052
In	1	Cl	-1	8	0.413	2.4386	6.00	0.043	8.50	2.4455	3.93	0.048	8.63	2.5318	3.99	0.059
In	1	I	-1	6	0.514	2.5077	7.00	0.017	7.83	2.5122	4.31	0.017	7.83	2.7987	4.22	0.032
In	1	Te	-2	3	0.508	2.4779	6.50	0.057	7.67	2.4837	4.25	0.059	7.67	2.7572	4.17	0.127
In	3	As	-3	7	0.553	2.5433	6.50	0.065	4.00	2.5516	3.51	0.065	4.00	2.6037	3.37	0.097
In	3	Br	-1	5	0.589	2.2894	6.50	0.083	4.40	2.3201	3.40	0.132	4.40	2.4012	3.21	0.087
In	3	Cl	-1	4	0.548	2.1347	6.00	0.017	6.00	2.1422	3.31	0.012	6.00	2.2627	3.18	0.038
In	3	F	-1	17	0.421	1.7573	5.50	0.072	6.47	1.7679	2.70	0.089	6.53	1.8061	2.76	0.081
In	3	I	-1	5	0.667	2.4777	7.00	0.012	4.00	2.5279	3.68	0.047	4.00	2.6120	3.38	0.074
In	3	N	-3	2	0.416	2.0234	6.00	0.012	4.00	2.0358	2.76	0.012	4.00	2.0491	2.82	0.014
In	3	O	-2	41	0.353	1.9030	5.00	0.145	6.00	1.9096	2.67	0.150	6.02	1.8979	2.82	0.142
In	3	P	-3	6	0.446	2.5097	6.00	0.040	3.83	2.5105	3.27	0.040	3.83	2.5284	3.28	0.083
In	3	Sb	-3	5	0.634	2.6439	7.00	0.095	4.00	2.6799	3.78	0.059	4.00	2.7551	3.52	0.101
In	3	Se	-2	20	0.484	2.4305	6.00	0.101	4.30	2.4450	3.32	0.100	4.30	2.4842	3.28	0.170
In	3	S	-2	75	0.456	2.3080	6.00	0.103	5.45	2.3192	3.25	0.107	5.45	2.3676	3.25	0.142
In	3	Te	-2	15	0.534	2.6228	6.50	0.062	4.00	2.6485	3.57	0.064	4.00	2.6952	3.46	0.094
Ir	3	Cl	-1	2	0.402	2.0582	5.50	0.067	6.00	2.0627	2.92	0.067	6.00	2.0847	3.00	0.073
Ir	4	O	-2	12	0.436	1.8323	5.50	0.084	6.00	1.8394	2.65	0.077	6.00	1.8658	2.68	0.092
Ir	5	O	-2	4	0.479	1.8979	6.00	0.101	6.00	1.9050	2.69	0.108	6.00	1.9248	2.66	0.140

K	1	As	-3	38	0.674	2.3581	7.50	0.084	5.26	2.3946	4.49	0.086	4.84	2.8744	4.12	0.132
K	1	Br	-1	20	0.595	2.1721	7.00	0.051	10.00	2.1900	4.42	0.058	9.80	2.6898	4.20	0.135
K	1	Cl	-1	52	0.555	2.0767	6.50	0.049	9.04	2.0926	4.12	0.055	8.94	2.4908	3.96	0.093
K	1	F	-1	104	0.428	1.8333	5.50	0.056	9.80	1.8466	3.44	0.058	9.82	1.9748	3.48	0.059
K	1	H	-1	11	0.451	1.8764	5.50	0.115	9.09	1.8877	3.54	0.115	9.09	2.0630	3.54	0.120
K	1	I	-1	16	0.655	2.2888	7.00	0.050	8.44	2.3133	4.66	0.056	8.19	2.9020	4.34	0.126
K	1	N	-3	31	0.525	2.0009	6.00	0.056	7.00	2.0282	3.81	0.061	6.77	2.3150	3.69	0.110
K	1	Nx	-3	23	0.525	1.9663	6.00	0.050	6.48	1.9820	3.73	0.057	6.39	2.2629	3.61	0.095
K	1	O	-2	53	0.436	1.9423	6.00	0.091	8.81	1.9635	3.54	0.092	8.87	2.0989	3.57	0.098
K	1	P	-3	24	0.644	2.3738	7.00	0.087	5.04	2.4033	4.38	0.093	4.88	2.8284	4.08	0.127
K	1	Sb	-3	11	0.749	2.5472	8.00	0.072	4.27	2.5902	4.76	0.073	4.18	3.1177	4.31	0.144
K	1	Se	-2	53	0.604	2.2971	7.00	0.058	6.55	2.3225	4.33	0.060	6.36	2.7449	4.09	0.105
K	1	S	-2	40	0.573	2.1680	6.50	0.063	7.53	2.1943	4.18	0.062	7.23	2.5899	3.98	0.103
K	1	Te	-2	20	0.649	2.4180	7.00	0.053	6.40	2.4379	4.58	0.053	6.05	2.9406	4.27	0.084
La	3	Cl	-1	2	0.459	2.4310	6.00	0.059	7.50	2.4349	3.52	0.079	7.50	2.5116	3.51	0.258
La	3	F	-1	7	0.443	1.9658	5.50	0.036	10.29	1.9760	3.16	0.037	10.29	2.0608	3.18	0.029
La	3	O	-2	119	0.451	2.0639	5.50	0.100	9.27	2.0832	3.24	0.118	9.29	2.1679	3.25	0.135
La	3	S	-2	18	0.588	2.4033	6.50	0.078	7.94	2.4314	3.85	0.078	7.89	2.6362	3.66	0.135
Li	1	As	-3	13	0.754	1.5089	7.50	0.100	4.23	1.6030	3.78	0.091	4.15	2.1416	3.33	0.184
Li	1	Br	-1	13	0.675	1.5129	7.00	0.052	5.85	1.5674	3.74	0.048	5.85	2.1012	3.42	0.112
Li	1	Cl	-1	15	0.634	1.3989	6.50	0.052	5.87	1.4565	3.50	0.052	5.87	1.9193	3.24	0.072
Li	1	F	-1	52	0.508	1.0867	5.50	0.044	5.56	1.1426	2.75	0.052	5.56	1.3734	2.67	0.061
Li	1	H	-1	7	0.531	1.0390	5.50	0.017	7.71	1.0818	2.93	0.035	7.71	1.3937	2.81	0.097
Li	1	I	-1	11	0.735	1.6483	7.00	0.049	5.82	1.7061	4.06	0.046	5.82	2.3415	3.66	0.126
Li	1	N	-3	45	0.637	1.1543	6.00	0.078	4.16	1.2393	3.07	0.080	4.07	1.6121	2.79	0.114
Li	1	Nx	-3	11	0.605	1.1235	6.00	0.008	6.00	1.1236	3.08	0.008	6.00	1.5446	2.87	0.014
Li	1	O	-2	96	0.516	1.1710	5.50	0.040	5.07	1.2316	2.82	0.061	5.03	1.4614	2.72	0.072
Li	1	P	-3	6	0.724	1.4786	7.00	0.053	4.17	1.5673	3.65	0.071	4.17	2.0648	3.26	0.088
Li	1	Sb	-3	3	0.821	1.6902	8.00	0.032	4.33	1.7962	4.19	0.043	4.33	2.6201	3.83	0.852
Li	1	Se	-2	6	0.684	1.6027	7.00	0.109	4.33	1.6562	3.65	0.092	4.33	2.1113	3.32	0.147

Li	1	S	-2	13	0.653	1.4665	6.00	0.040	4.38	1.5516	3.46	0.071	4.38	1.9613	3.17	0.075
Li	1	Te	-2	7	0.729	1.7103	7.00	0.074	5.14	1.7726	4.02	0.073	5.14	2.3449	3.61	0.195
Lu	3	Cl	-1	4	0.433	2.2840	6.00	0.033	6.00	2.2934	3.22	0.047	6.00	2.3371	3.26	0.055
Lu	3	F	-1	2	0.412	1.8074	5.50	0.143	7.00	1.8122	2.76	0.144	7.00	1.8451	2.82	0.175
Lu	3	O	-2	47	0.421	1.9173	5.50	0.129	6.77	1.9295	2.88	0.127	6.77	1.9697	2.93	0.149
Lu	3	S	-2	13	0.557	2.2812	7.00	0.079	6.08	2.3053	3.50	0.068	6.08	2.4362	3.36	0.126
Mg	2	As	-3	3	0.750	2.0440	8.00	0.111	4.67	2.1268	3.85	0.073	4.67	2.4371	3.41	0.314
Mg	2	Br	-1	3	0.671	1.8906	7.00	0.032	6.00	1.9399	3.65	0.020	6.00	2.2701	3.34	0.035
Mg	2	Cl	-1	9	0.630	1.7828	6.50	0.046	6.00	1.8064	3.41	0.052	6.00	2.0916	3.16	0.086
Mg	2	F	-1	21	0.504	1.4096	5.50	0.108	6.00	1.4337	2.72	0.083	6.00	1.5805	2.65	0.112
Mg	2	H	-1	20	0.559	1.2869	5.50	0.084	8.30	1.3378	2.94	0.090	8.30	1.5892	2.78	0.280
Mg	2	Hx	-1	3	0.527	1.2747	5.50	0.103	4.67	1.2951	2.50	0.191	4.33	1.4056	2.35	0.443
Mg	2	N	-3	7	0.601	1.6352	6.00	0.058	4.14	1.6790	2.99	0.075	4.14	1.8379	2.77	0.142
Mg	2	O	-2	87	0.512	1.4840	5.50	0.078	5.90	1.5381	2.83	0.083	5.90	1.6895	2.75	0.091
Mg	2	P	-3	3	0.563	2.1310	7.50	0.116	4.33	2.1740	3.42	0.073	4.33	2.3201	3.27	0.185
Mg	2	Sb	-3	5	0.634	2.3927	8.00	0.063	4.40	2.4248	3.84	0.046	4.40	2.6269	3.58	0.205
Mg	2	S	-2	9	0.649	1.8237	6.50	0.093	5.56	1.8844	3.49	0.116	5.56	2.1631	3.20	0.209
Mn	2	As	-3	10	0.567	2.1709	6.50	0.084	4.10	2.1969	3.42	0.069	4.10	2.3370	3.27	0.127
Mn	2	Br	-1	9	0.528	2.0898	6.00	0.054	5.78	2.0952	3.42	0.065	5.78	2.2601	3.32	0.184
Mn	2	Cl	-1	21	0.488	1.9968	6.00	0.056	5.90	2.0023	3.24	0.060	5.90	2.1289	3.19	0.092
Mn	2	F	-1	33	0.474	1.5649	5.50	0.040	6.12	1.5889	2.80	0.062	6.12	1.7041	2.78	0.078
Mn	2	I	-1	4	0.588	2.2605	6.50	0.020	6.00	2.2719	3.77	0.009	6.00	2.5106	3.58	0.018
Mn	2	N	-3	6	0.606	1.4734	6.50	0.080	4.33	1.6058	2.95	0.153	4.33	1.7838	2.73	0.207
Mn	2	O	-2	155	0.481	1.6276	5.50	0.074	5.95	1.6665	2.89	0.080	5.94	1.7855	2.85	0.098
Mn	2	Se	-2	10	0.431	2.2475	5.50	0.094	4.40	2.2523	3.22	0.081	4.40	2.2993	3.25	0.102
Mn	2	S	-2	26	0.406	2.1547	5.50	0.075	5.31	2.1589	3.14	0.073	5.31	2.1931	3.22	0.095
Mn	2	Te	-2	6	0.470	2.4245	6.00	0.069	4.33	2.4275	3.47	0.062	4.33	2.5037	3.45	0.037
Mn	3	F	-1	30	0.446	1.6018	5.50	0.073	6.00	1.6114	2.57	0.067	6.00	1.6609	2.58	0.081
Mn	3	O	-2	65	0.437	1.6899	5.50	0.095	5.86	1.7066	2.63	0.095	5.86	1.7484	2.66	0.105
Mn	4	Cl	-1	2	0.517	2.0654	6.50	0.027	6.00	2.0694	3.03	0.023	6.00	2.1290	2.94	0.032

Mn	4	F	-1	4	0.410	1.6161	5.00	0.181	6.00	1.6202	2.38	0.213	6.00	1.6360	2.45	0.231
Mn	4	O	-2	26	0.402	1.7327	5.00	0.112	5.92	1.7408	2.48	0.116	5.92	1.7531	2.56	0.124
Mn	5	O	-2	8	0.413	1.7888	5.50	0.043	4.00	1.7927	2.30	0.047	4.00	1.7831	2.36	0.052
Mn	6	O	-2	4	0.416	1.8202	5.50	0.078	4.00	1.8226	2.26	0.070	4.00	1.8039	2.32	0.078
Mn	7	O	-2	11	0.520	1.8736	6.50	0.078	4.00	1.9055	2.37	0.149	4.00	1.8207	2.28	0.204
Mo	2	Br	-1	2	0.401	2.2222	5.50	0.008	5.00	2.2267	3.17	0.008	5.00	2.2551	3.26	0.009
Mo	2	Cl	-1	9	0.441	2.0518	5.50	0.042	5.00	2.0609	3.10	0.044	5.00	2.1258	3.13	0.053
Mo	2	I	-1	4	0.461	2.3570	6.00	0.013	5.00	2.3658	3.46	0.014	5.00	2.4488	3.45	0.017
Mo	2	S	-2	5	0.422	2.0717	5.50	0.047	5.00	2.0824	3.08	0.050	5.00	2.1295	3.13	0.057
Mo	3	Br	-1	4	0.541	2.1915	6.00	0.115	6.00	2.1996	3.36	0.092	6.00	2.3176	3.24	0.134
Mo	3	Cl	-1	7	0.501	2.0894	6.00	0.031	6.00	2.0978	3.17	0.046	6.00	2.1881	3.11	0.062
Mo	3	F	-1	8	0.427	1.7382	5.50	0.057	6.00	1.7412	2.66	0.056	6.00	1.7807	2.70	0.064
Mo	3	O	-2	20	0.418	1.7893	5.50	0.039	5.70	1.8042	2.68	0.053	5.70	1.8345	2.73	0.048
Mo	3	S	-2	8	0.519	2.0626	6.00	0.040	6.00	2.0962	3.21	0.042	6.00	2.1963	3.12	0.057
Mo	4	Cl	-1	4	0.558	2.1283	6.50	0.070	6.00	2.1498	3.18	0.042	6.00	2.2238	3.04	0.101
Mo	4	O	-2	28	0.562	1.7239	6.50	0.086	6.00	1.7792	2.82	0.097	6.00	1.8546	2.67	0.146
Mo	5	O	-2	50	0.482	1.8476	5.50	0.062	5.96	1.8690	2.65	0.077	5.88	1.8788	2.60	0.124
Mo	6	O	-2	246	0.391	1.9093	5.00	0.106	4.63	1.9196	2.38	0.144	4.73	1.9113	2.49	0.139
N	3	N	-3	8	0.537	1.7255	6.00	0.098	1.00	1.7633	1.95	0.110	1.00	1.5797	1.84	0.160
N	3	O	-2	35	0.448	1.4079	5.00	0.044	2.00	1.4241	1.89	0.048	2.00	1.3924	1.91	0.058
N	5	F	-1	1	0.550	1.4072	6.00	0.000	4.00	1.4577	2.13	0.000	4.00	1.4176	2.00	0.000
N	5	N	-3	1	0.532	1.6839	6.00	0.000	2.00	1.6945	1.98	0.000	2.00	1.5460	1.87	0.000
N	5	O	-2	70	0.450	1.4627	5.00	0.056	3.00	1.4747	1.90	0.071	3.00	1.4337	1.91	0.086
Na	1	As	-3	38	0.720	1.9358	7.50	0.079	4.89	1.9971	4.19	0.078	4.55	2.5274	3.75	0.166
Na	1	Br	-1	9	0.641	1.7823	7.00	0.024	6.00	1.8211	3.90	0.024	6.00	2.3052	3.63	0.041
Na	1	Cl	-1	21	0.601	1.6949	6.50	0.029	6.62	1.7394	3.74	0.036	6.62	2.1633	3.53	0.060
Na	1	F	-1	51	0.474	1.4288	5.50	0.031	7.14	1.4658	3.08	0.037	7.10	1.6640	3.05	0.050
Na	1	H	-1	5	0.497	1.4497	5.50	0.113	7.60	1.4769	3.20	0.104	7.60	1.7299	3.14	0.128
Na	1	I	-1	4	0.701	1.9439	7.00	0.018	6.00	1.9788	4.25	0.009	6.00	2.5633	3.89	0.056
Na	1	N	-3	35	0.571	1.6073	6.00	0.056	5.20	1.6609	3.43	0.060	5.00	1.9765	3.23	0.110

Na	1	Nx	-3	4	0.571	1.4822	6.00	0.049	6.00	1.4948	3.34	0.053	6.00	1.8536	3.18	0.083
Na	1	O	-2	125	0.482	1.5622	6.00	0.087	5.90	1.6027	3.16	0.091	5.76	1.7941	3.10	0.110
Na	1	P	-3	32	0.690	1.8958	7.00	0.059	4.84	1.9531	4.04	0.061	4.56	2.4382	3.66	0.133
Na	1	Sb	-3	10	0.795	1.9877	8.00	0.102	5.10	2.0590	4.50	0.097	4.50	2.7093	3.93	0.214
Na	1	Se	-2	70	0.650	1.8988	7.00	0.053	5.87	1.9415	4.03	0.055	5.60	2.4157	3.72	0.090
Na	1	S	-2	93	0.619	1.8314	6.50	0.085	5.76	1.8708	3.85	0.085	5.58	2.2931	3.59	0.118
Na	1	Te	-2	46	0.695	2.0301	7.00	0.067	5.87	2.0671	4.30	0.063	5.74	2.6272	3.94	0.088
Nb	2	Te	-2	5	0.448	2.3909	6.00	0.049	6.00	2.3981	3.54	0.053	6.00	2.4788	3.55	0.045
Nb	3	O	-2	4	0.501	1.7458	6.00	0.011	6.00	1.7843	2.86	0.033	6.00	1.8751	2.79	0.045
Nb	4	F	-1	5	0.534	1.7584	6.00	0.055	6.00	1.8044	2.79	0.136	6.00	1.8708	2.68	0.197
Nb	4	O	-2	12	0.526	1.7854	6.00	0.074	6.00	1.8318	2.81	0.093	6.00	1.8922	2.71	0.131
Nb	4	S	-2	8	0.412	2.2801	5.50	0.038	5.75	2.2878	3.03	0.037	5.75	2.3027	3.10	0.087
Nb	5	As	-3	2	0.640	2.6648	7.00	0.039	4.00	2.6683	3.45	0.033	4.00	2.6077	3.19	0.057
Nb	5	Cl	-1	10	0.616	2.2099	6.50	0.050	6.00	2.2382	3.24	0.059	6.00	2.2778	3.01	0.086
Nb	5	F	-1	6	0.489	1.7603	5.50	0.085	6.00	1.7769	2.57	0.068	6.00	1.7950	2.53	0.097
Nb	5	N	-3	5	0.480	2.0288	6.00	0.225	4.40	2.0388	2.67	0.232	4.40	2.0225	2.64	0.375
Nb	5	O	-2	160	0.498	1.8659	5.50	0.102	6.04	1.8904	2.71	0.115	6.04	1.9086	2.64	0.130
Nd	3	F	-1	2	0.437	1.9110	5.50	0.006	10.00	1.9200	3.08	0.015	10.00	1.9967	3.11	0.055
Nd	3	O	-2	85	0.428	2.0243	5.50	0.100	8.21	2.0404	3.09	0.121	8.35	2.0952	3.14	0.127
Nd	3	S	-2	10	0.510	2.4462	6.00	0.108	7.70	2.4587	3.68	0.097	7.70	2.5883	3.60	0.142
Nh[#3]	1	Br	-1	13	0.601	2.2328	6.50	0.071	9.69	2.2426	4.48	0.065	9.69	2.7571	4.26	0.138
Nh	1	Cl	-1	33	0.561	2.1536	6.50	0.069	9.42	2.1691	4.24	0.074	9.33	2.5874	4.08	0.105
Nh	1	F	-1	59	0.434	1.9793	5.50	0.070	10.10	1.9953	3.63	0.073	10.17	2.1352	3.66	0.077
Nh	1	I	-1	11	0.661	2.4058	7.00	0.038	6.91	2.4197	4.65	0.042	6.91	2.9749	4.35	0.071
Nh	1	O	-2	92	0.442	2.0338	6.00	0.066	9.59	2.0617	3.70	0.072	9.64	2.2154	3.72	0.069
Nh	1	S	-2	11	0.579	2.2426	7.00	0.033	8.18	2.2687	4.32	0.029	8.00	2.6904	4.12	0.097
Ni	2	Br	-1	2	0.516	1.9673	6.00	0.105	6.00	1.9785	3.29	0.130	6.00	2.1385	3.21	0.181
Ni	2	Cl	-1	4	0.476	1.8767	5.50	0.036	6.00	1.8829	3.09	0.054	6.00	1.9990	3.07	0.070
Ni	2	F	-1	45	0.452	1.4963	5.00	0.080	6.00	1.5097	2.66	0.064	6.00	1.5996	2.67	0.078
Ni	2	O	-2	135	0.443	1.5592	5.50	0.080	5.93	1.5793	2.70	0.085	5.93	1.6575	2.72	0.099

Ni	2	P	-3	3	0.323	2.0689	6.50	0.009	4.00	2.0709	2.76	0.008	4.00	2.0383	2.96	0.007
Ni	2	Se	-2	4	0.525	1.8986	6.00	0.081	5.25	1.9321	3.20	0.092	5.25	2.0795	3.10	0.106
Ni	2	S	-2	10	0.494	1.8570	5.50	0.077	4.70	1.8729	3.01	0.045	4.70	1.9762	2.96	0.117
Ni	2	Te	-2	5	0.571	2.1118	6.50	0.019	4.00	2.1601	3.38	0.022	4.00	2.2989	3.22	0.032
Ni	3	F	-1	5	0.423	1.5687	5.00	0.071	6.00	1.5744	2.48	0.056	6.00	1.6111	2.53	0.064
Ni	3	O	-2	15	0.414	1.6489	5.50	0.058	6.00	1.6565	2.54	0.072	6.00	1.6870	2.61	0.080
Ni	3	S	-2	4	0.523	1.9212	5.50	0.185	5.00	1.9615	2.99	0.263	5.00	2.0382	2.89	0.129
Ni	4	F	-1	7	0.411	1.6145	5.50	0.117	6.00	1.6197	2.38	0.111	6.00	1.6363	2.45	0.123
Ni	4	O	-2	4	0.402	1.7216	5.00	0.202	6.00	1.7332	2.48	0.285	6.00	1.7454	2.56	0.316
Np	4	O	-2	3	0.531	1.9458	6.00	0.083	7.33	1.9668	3.06	0.105	7.33	2.0620	2.95	0.307
Np	5	O	-2	5	0.431	2.0304	5.50	0.101	7.00	2.0367	2.81	0.097	7.00	2.0390	2.83	0.112
Np	6	O	-2	3	0.500	2.0157	6.00	0.151	7.00	2.0381	2.84	0.202	7.00	2.0284	2.75	0.326
Os	4	Br	-1	2	0.461	2.3061	6.00	0.157	6.00	2.3111	3.17	0.128	6.00	2.3472	3.16	0.167
Os	4	Cl	-1	4	0.421	2.1557	6.00	0.031	6.00	2.1603	2.94	0.038	6.00	2.1808	2.99	0.042
Os	4	O	-2	3	0.498	1.7530	6.00	0.124	6.00	1.7865	2.71	0.178	6.00	1.8376	2.65	0.233
Os	5	F	-1	3	0.488	1.7581	6.00	0.340	6.00	1.7790	2.57	0.401	6.00	1.7979	2.53	0.500
Os	7	O	-2	3	0.479	1.9578	5.50	0.060	6.00	1.9657	2.58	0.043	6.00	1.9465	2.55	0.109
Os	8	O	-2	5	0.512	1.9726	6.00	0.102	5.40	1.9956	2.54	0.096	5.40	1.9295	2.45	0.251
P	3	O	-2	25	0.402	1.5153	4.50	0.020	3.00	1.5223	2.10	0.022	3.00	1.5223	2.19	0.024
P	3	S	-2	2	0.536	1.9609	6.00	0.036	3.00	2.0218	2.80	0.033	3.00	2.0218	2.68	0.047
P	5	Cl	-1	13	0.662	2.0109	7.00	0.093	4.00	2.0649	2.87	0.082	4.00	1.9993	2.58	0.149
P	5	F	-1	3	0.535	1.4761	7.00	0.020	6.00	1.4917	2.36	0.083	6.00	1.5218	2.25	0.120
P	5	I	-1	1	0.763	2.4979	7.50	0.000	4.00	2.5656	3.50	0.000	4.00	2.4777	3.06	0.000
P	5	N	-3	14	0.517	1.7327	6.00	0.119	4.00	1.7576	2.39	0.107	4.00	1.7244	2.30	0.145
P	5	O	-2	252	0.437	1.6204	5.00	0.057	4.00	1.6323	2.17	0.059	4.00	1.6169	2.20	0.070
P	5	Se	-2	12	0.600	2.3208	7.00	0.117	4.00	2.3443	3.08	0.108	4.00	2.2924	2.87	0.175
P	5	S	-2	23	0.566	2.1492	6.00	0.044	4.00	2.1701	2.86	0.051	4.00	2.1255	2.71	0.075
Pb	2	Br	-1	7	0.515	2.4316	6.00	0.074	7.43	2.4429	3.86	0.051	7.14	2.6240	3.76	0.119
Pb	2	Cl	-1	6	0.474	2.3464	5.50	0.044	7.33	2.3574	3.66	0.026	7.33	2.4863	3.63	0.058
Pb	2	F	-1	34	0.453	1.9092	6.00	0.061	9.18	1.9332	3.28	0.077	9.18	2.0501	3.28	0.116

Pb	2	I	-1	22	0.575	2.5673	7.00	0.032	6.00	2.5840	4.05	0.021	6.00	2.8086	3.88	0.031
Pb	2	N	-3	1	0.445	2.0988	6.00	0.000	5.00	2.1419	3.19	0.000	5.00	2.2045	3.21	0.000
Pb	2	O	-2	98	0.433	2.0192	5.50	0.093	7.51	2.0461	3.25	0.117	7.62	2.1185	3.28	0.168
Pb	2	Se	-2	7	0.524	2.4676	6.50	0.066	7.14	2.4842	3.91	0.047	7.14	2.6725	3.81	0.124
Pb	2	S	-2	49	0.493	2.3876	6.00	0.074	7.43	2.4015	3.76	0.079	7.35	2.5498	3.69	0.101
Pb	4	F	-1	4	0.424	1.9343	5.50	0.060	6.00	1.9488	2.73	0.033	6.00	1.9707	2.78	0.038
Pb	4	O	-2	27	0.354	2.0329	5.00	0.235	5.70	2.0354	2.67	0.236	5.70	2.0299	2.82	0.237
Pd	1	As	-3	15	0.532	1.8197	6.50	0.061	3.60	1.8433	3.29	0.070	3.60	2.0469	3.18	0.081
Pd	2	As	-3	5	0.639	2.0411	7.00	0.071	4.00	2.0999	3.47	0.053	4.00	2.2862	3.21	0.091
Pd	2	Br	-1	6	0.462	2.1018	5.50	0.087	4.00	2.1192	3.11	0.058	4.00	2.1829	3.10	0.073
Pd	2	Cl	-1	16	0.421	1.9982	5.00	0.072	4.00	2.0102	2.91	0.055	4.00	2.0455	2.96	0.063
Pd	2	F	-1	29	0.506	1.5688	5.50	0.048	5.17	1.6063	2.82	0.073	5.17	1.7317	2.75	0.180
Pd	2	O	-2	44	0.498	1.6236	5.50	0.102	4.00	1.6687	2.73	0.093	4.00	1.7571	2.68	0.124
Pd	2	P	-3	3	0.641	1.9529	7.00	0.052	4.00	2.0239	3.40	0.072	4.00	2.2115	3.13	0.124
Pd	2	Se	-2	8	0.471	2.1344	6.00	0.034	4.00	2.1395	3.15	0.032	4.00	2.2094	3.13	0.040
Pd	2	S	-2	10	0.439	2.0256	5.50	0.113	4.00	2.0386	2.98	0.102	4.00	2.0863	3.01	0.121
Pd	4	Cl	-1	2	0.470	2.1266	6.00	0.063	6.00	2.1289	3.00	0.064	6.00	2.1694	2.98	0.081
Pd	4	F	-1	3	0.457	1.6858	5.50	0.132	6.00	1.6952	2.54	0.130	6.00	1.7304	2.54	0.161
Pd	4	O	-2	3	0.449	1.8050	5.50	0.078	5.33	1.8243	2.60	0.110	5.33	1.8457	2.62	0.034
Pd	4	Se	-2	2	0.520	2.3111	6.50	0.199	6.00	2.3396	3.30	0.373	5.00	2.3949	3.14	0.468
Pr	3	Cl	-1	5	0.387	2.4862	5.50	0.034	7.40	2.4883	3.40	0.032	7.40	2.5034	3.50	0.037
Pr	3	F	-1	4	0.448	1.9175	5.50	0.039	9.00	1.9300	3.07	0.051	9.00	2.0126	3.08	0.055
Pr	3	O	-2	60	0.439	2.0365	5.50	0.097	8.83	2.0542	3.16	0.125	8.88	2.1233	3.19	0.140
Pr	3	Se	-2	3	0.529	2.5268	7.00	0.085	7.67	2.5423	3.80	0.097	7.67	2.6890	3.70	0.143
Pr	3	S	-2	4	0.499	2.4650	6.00	0.098	7.75	2.4797	3.68	0.076	7.75	2.5992	3.61	0.167
Pt	2	Cl	-1	5	0.456	1.9770	5.50	0.051	4.00	1.9889	2.96	0.018	4.00	2.0485	2.97	0.022
Pt	2	O	-2	7	0.463	1.6577	5.50	0.119	4.00	1.6842	2.68	0.087	4.00	1.7486	2.67	0.109
Pt	2	Se	-2	6	0.407	2.1652	6.00	0.041	4.00	2.1699	3.04	0.038	4.00	2.1955	3.11	0.042
Pt	2	S	-2	8	0.438	2.0245	5.50	0.104	4.00	2.0333	2.97	0.087	4.00	2.0803	3.00	0.104
Pt	3	O	-2	3	0.546	1.6656	5.50	0.070	5.00	1.7506	2.82	0.046	5.00	1.8394	2.69	0.067

Pt	4	Cl	-1	5	0.440	2.1411	6.00	0.070	6.00	2.1423	2.96	0.070	6.00	2.1707	2.98	0.084
Pt	4	F	-1	15	0.488	1.6909	5.50	0.113	6.00	1.7137	2.62	0.132	6.00	1.7612	2.57	0.170
Pt	4	O	-2	18	0.479	1.8220	5.50	0.131	6.00	1.8369	2.72	0.100	6.00	1.8810	2.69	0.130
Pt	4	Se	-2	5	0.489	2.3094	6.00	0.046	6.00	2.3119	3.22	0.036	6.00	2.3601	3.17	0.047
Pt	4	S	-2	3	0.458	2.2000	6.00	0.306	6.00	2.2085	3.06	0.319	6.00	2.2401	3.05	0.341
Pt	5	F	-1	3	0.424	1.7857	5.50	0.204	6.00	1.7972	2.49	0.204	6.00	1.8058	2.54	0.243
Pu	3	Cl	-1	3	0.548	2.2769	7.00	0.062	7.67	2.2906	3.60	0.058	7.67	2.4545	3.46	0.212
Pu	4	O	-2	3	0.363	2.0794	5.50	0.027	7.33	2.0821	2.83	0.026	7.33	2.0779	2.97	0.022
Rb	1	As	-3	11	0.650	2.5443	7.50	0.055	5.27	2.5703	4.59	0.063	5.09	3.0147	4.28	0.103
Rb	1	Br	-1	31	0.572	2.3436	7.00	0.072	9.77	2.3563	4.49	0.075	9.74	2.8029	4.31	0.135
Rb	1	Cl	-1	67	0.531	2.2658	6.50	0.056	9.73	2.2756	4.25	0.055	9.70	2.6313	4.14	0.107
Rb	1	F	-1	15	0.404	1.9914	5.50	0.051	10.47	2.0019	3.54	0.046	10.47	2.0797	3.61	0.049
Rb	1	H	-1	5	0.428	2.0508	5.50	0.077	8.80	2.0579	3.61	0.078	8.80	2.1833	3.65	0.062
Rb	1	I	-1	23	0.632	2.4644	7.00	0.071	9.00	2.4826	4.79	0.066	8.78	3.0366	4.50	0.150
Rb	1	N	-3	12	0.501	2.1922	6.00	0.069	8.42	2.2051	4.00	0.071	8.33	2.4713	3.92	0.103
Rb	1	Nx	-3	13	0.501	2.1499	6.00	0.027	6.31	2.1576	3.81	0.029	6.31	2.3939	3.74	0.034
Rb	1	O	-2	44	0.413	2.0860	6.50	0.056	9.77	2.1029	3.64	0.054	9.93	2.1959	3.71	0.061
Rb	1	P	-3	10	0.621	2.5292	7.00	0.034	5.40	2.5481	4.49	0.044	5.40	2.9558	4.24	0.073
Rb	1	Sb	-3	6	0.725	2.6941	8.00	0.047	5.00	2.7249	4.94	0.058	5.00	3.2731	4.53	0.186
Rb	1	Se	-2	19	0.580	2.4036	7.00	0.097	7.32	2.4376	4.42	0.110	7.26	2.8399	4.24	0.130
Rb	1	S	-2	29	0.550	2.3082	6.50	0.048	6.48	2.3274	4.15	0.050	6.48	2.6596	4.01	0.076
Rb	1	Te	-2	3	0.626	2.4346	7.50	0.016	9.67	2.4601	4.79	0.011	9.00	3.0186	4.49	0.068
Re	3	Br	-1	4	0.422	2.3042	5.50	0.019	4.75	2.3233	3.13	0.039	4.75	2.3457	3.18	0.021
Re	3	Cl	-1	5	0.420	2.1578	5.50	0.022	5.00	2.1768	3.00	0.022	5.00	2.2011	3.05	0.025
Re	3	S	-2	10	0.401	2.2071	6.00	0.025	5.00	2.2133	3.00	0.029	5.00	2.2290	3.08	0.031
Re	4	Cl	-1	7	0.412	2.1851	5.50	0.057	6.00	2.1875	2.95	0.049	6.00	2.2043	3.02	0.057
Re	4	O	-2	4	0.507	1.7824	6.00	0.012	6.00	1.8162	2.76	0.044	6.00	1.8709	2.68	0.067
Re	5	O	-2	5	0.479	1.8266	6.00	0.115	6.00	1.8533	2.63	0.077	6.00	1.8655	2.60	0.212
Re	6	O	-2	4	0.498	1.9101	6.00	0.081	5.50	1.9321	2.61	0.228	5.50	1.9171	2.55	0.566
Re	7	O	-2	41	0.508	1.9779	6.00	0.147	4.10	2.0053	2.47	0.192	4.10	1.9302	2.40	0.225

Rh	3	Cl	-1	2	0.440	2.0372	5.00	0.004	6.00	2.0378	2.98	0.003	6.00	2.0863	3.01	0.004
Rh	3	F	-1	8	0.488	1.6369	5.50	0.096	6.00	1.6457	2.69	0.052	6.00	1.7275	2.65	0.069
Rh	3	O	-2	13	0.478	1.6701	5.50	0.133	6.00	1.7003	2.72	0.131	6.00	1.7749	2.69	0.167
Rh	4	F	-1	1	0.406	1.6869	5.50	0.000	6.00	1.6924	2.44	0.000	6.00	1.7070	2.52	0.000
Rh	4	O	-2	1	0.403	1.7768	5.50	0.000	6.00	1.7924	2.54	0.000	6.00	1.8056	2.62	0.000
Ru	3	Cl	-1	5	0.489	1.9952	5.50	0.073	6.00	2.0045	3.05	0.057	6.00	2.0868	3.01	0.074
Ru	3	O	-2	5	0.430	1.7207	5.50	0.072	6.00	1.7337	2.65	0.058	6.00	1.7752	2.69	0.067
Ru	4	O	-2	20	0.449	1.7936	5.50	0.057	6.00	1.8055	2.64	0.064	6.00	1.8372	2.65	0.079
Ru	5	F	-1	7	0.445	1.7763	5.50	0.344	6.00	1.7850	2.51	0.341	6.00	1.7982	2.53	0.414
Ru	5	O	-2	9	0.436	1.8744	5.50	0.041	6.00	1.8785	2.59	0.038	6.00	1.8901	2.62	0.040
Ru	6	O	-2	4	0.425	1.9258	5.50	0.069	4.50	1.9275	2.42	0.060	4.50	1.9105	2.47	0.081
S	3	N	-3	4	0.432	1.7701	6.00	0.032	2.00	1.8022	2.25	0.023	2.00	1.7771	2.29	0.027
S	4	Cl	-1	6	0.545	2.0527	6.50	0.027	3.00	2.1296	2.76	0.049	3.00	2.0792	2.64	0.073
S	4	N	-3	1	0.515	1.8108	6.00	0.000	2.00	1.8394	2.23	0.000	2.00	1.7389	2.15	0.000
S	4	O	-2	18	0.427	1.6428	5.50	0.066	3.00	1.6546	2.15	0.070	3.00	1.6381	2.19	0.081
S	6	O	-2	195	0.441	1.6422	5.00	0.082	4.00	1.6511	2.11	0.085	4.00	1.6221	2.14	0.100
Sb	3	Br	-1	4	0.537	2.3817	7.00	0.072	4.75	2.4213	3.45	0.266	4.75	2.4877	3.32	0.113
Sb	3	Cl	-1	5	0.496	2.3083	6.00	0.159	4.60	2.3334	3.26	0.123	4.40	2.3774	3.18	0.232
Sb	3	F	-1	18	0.431	1.8345	5.50	0.026	4.72	1.8660	2.69	0.122	4.94	1.8853	2.73	0.083
Sb	3	I	-1	5	0.597	2.5991	7.50	0.046	6.00	2.6188	3.90	0.102	5.80	2.7432	3.65	0.113
Sb	3	O	-2	24	0.423	1.9204	5.00	0.070	4.54	1.9488	2.78	0.148	4.54	1.9727	2.79	0.148
Sb	3	Se	-2	14	0.546	2.4759	6.50	0.070	5.29	2.5122	3.61	0.101	4.71	2.5897	3.42	0.209
Sb	3	S	-2	104	0.515	2.3695	6.00	0.090	4.87	2.4050	3.40	0.133	4.45	2.4551	3.26	0.158
Sb	3	Te	-2	3	0.591	2.7314	7.00	0.116	4.00	2.7894	3.81	0.013	4.00	2.8333	3.60	0.553
Sb	5	Cl	-1	5	0.616	2.2232	6.50	0.072	6.00	2.2446	3.25	0.059	6.00	2.2890	3.02	0.107
Sb	5	F	-1	20	0.489	1.7660	6.00	0.062	6.00	1.7833	2.58	0.058	6.00	1.8045	2.53	0.075
Sb	5	O	-2	51	0.400	1.8977	5.50	0.111	6.00	1.9057	2.56	0.105	6.00	1.9110	2.64	0.113
Sb	5	Se	-2	2	0.551	2.5898	7.00	0.003	4.00	2.5938	3.27	0.010	4.00	2.5534	3.13	0.016
Sb	5	S	-2	5	0.520	2.4425	6.00	0.045	4.00	2.4536	3.09	0.075	4.00	2.4200	3.00	0.104
Sc	3	Cl	-1	5	0.612	2.1133	6.50	0.145	6.00	2.1424	3.45	0.145	6.00	2.3078	3.23	0.262

Sc	3	F	-1	12	0.485	1.6532	6.00	0.042	6.17	1.6635	2.71	0.067	6.17	1.7459	2.68	0.107
Sc	3	O	-2	51	0.494	1.7322	5.50	0.098	6.25	1.7581	2.84	0.109	6.25	1.8473	2.78	0.138
Sc	3	S	-2	21	0.631	2.0945	7.00	0.056	6.05	2.1423	3.50	0.060	6.05	2.3244	3.25	0.118
Se	4	Br	-1	5	0.585	2.3272	7.00	0.032	6.00	2.3496	3.43	0.030	5.40	2.3972	3.17	0.082
Se	4	Cl	-1	17	0.545	2.1585	6.50	0.048	6.00	2.1769	3.19	0.045	5.59	2.2141	3.00	0.145
Se	4	O	-2	161	0.427	1.8009	5.50	0.069	3.00	1.8271	2.32	0.092	3.00	1.8105	2.37	0.104
Se	6	O	-2	37	0.416	1.7987	5.50	0.093	4.00	1.8056	2.24	0.100	4.00	1.7868	2.30	0.111
Si	2	I	-1	7	0.578	2.3228	6.50	0.033	2.00	2.4290	3.27	0.026	2.00	2.4290	3.09	0.040
Si	4	As	-3	6	0.340	2.3877	6.00	0.070	3.67	2.3878	2.85	0.070	3.67	2.3907	3.02	0.082
Si	4	F	-1	6	0.529	1.4565	5.50	0.041	6.00	1.4681	2.45	0.043	6.00	1.5325	2.35	0.062
Si	4	N	-3	47	0.455	1.7120	6.00	0.095	4.00	1.7374	2.40	0.095	4.00	1.7369	2.40	0.117
Si	4	O	-2	220	0.432	1.6082	5.00	0.097	4.10	1.6233	2.26	0.116	4.10	1.6244	2.30	0.136
Si	4	P	-3	4	0.404	2.2567	6.50	0.114	4.00	2.2591	2.84	0.097	4.00	2.2591	2.92	0.106
Si	4	Se	-2	8	0.593	2.2355	6.50	0.043	4.00	2.2645	3.12	0.052	4.00	2.2639	2.93	0.088
Si	4	S	-2	19	0.560	2.0998	6.50	0.096	4.00	2.1248	2.94	0.106	4.00	2.1241	2.79	0.157
Sm	3	F	-1	7	0.441	1.8884	5.50	0.037	8.43	1.9014	3.00	0.038	8.43	1.9738	3.02	0.041
Sm	3	O	-2	42	0.433	2.0117	5.50	0.118	7.81	2.0256	3.07	0.126	7.81	2.0831	3.10	0.140
Sm	3	S	-2	6	0.505	2.4146	6.00	0.106	7.67	2.4271	3.63	0.111	7.67	2.5505	3.56	0.150
Sm	3	Te	-2	1	0.581	2.7198	7.00	0.000	6.00	2.7303	3.97	0.000	6.00	2.8765	3.80	0.000
Sn	2	As	-3	5	0.695	2.3837	7.00	0.015	3.00	2.4700	3.76	0.097	3.00	2.6010	3.41	0.183
Sn	2	Br	-1	2	0.501	2.3737	6.00	0.107	7.00	2.3753	3.73	0.114	7.00	2.5253	3.65	0.191
Sn	2	Cl	-1	5	0.461	2.2876	5.50	0.127	5.80	2.3070	3.46	0.185	5.60	2.3909	3.43	0.226
Sn	2	I	-1	10	0.562	2.5438	7.00	0.078	6.30	2.5554	4.01	0.097	6.30	2.7659	3.85	0.147
Sn	2	O	-2	34	0.458	1.8750	5.50	0.085	3.68	1.9212	2.86	0.213	3.65	1.9672	2.85	0.309
Sn	2	P	-3	2	0.550	2.2997	6.50	0.083	3.00	2.3282	3.35	0.094	3.00	2.4001	3.21	0.139
Sn	2	Sb	-3	1	0.655	2.5878	7.00	0.000	3.00	2.6174	3.83	0.000	3.00	2.7330	3.55	0.000
Sn	2	S	-2	15	0.479	2.3250	6.00	0.073	5.60	2.3554	3.54	0.090	5.27	2.4481	3.47	0.142
Sn	4	As	-3	5	0.522	2.5816	6.50	0.115	4.00	2.5988	3.35	0.100	4.00	2.5987	3.26	0.140
Sn	4	Cl	-1	9	0.588	2.1764	6.50	0.069	5.89	2.1904	3.27	0.105	5.89	2.2738	3.08	0.097
Sn	4	F	-1	18	0.461	1.7556	5.50	0.047	6.00	1.7701	2.62	0.060	6.00	1.8068	2.62	0.072

Sn	4	N	-3	3	0.449	1.9936	6.00	0.005	6.00	1.9953	2.83	0.004	6.00	2.0273	2.84	0.005
Sn	4	O	-2	29	0.379	1.8902	5.00	0.075	6.07	1.8959	2.60	0.074	6.07	1.8996	2.72	0.076
Sn	4	P	-3	3	0.523	2.5374	6.50	0.000	4.00	2.5484	3.31	0.027	4.00	2.5477	3.21	0.038
Sn	4	Se	-2	19	0.522	2.5073	6.00	0.057	4.11	2.5221	3.29	0.081	4.11	2.5241	3.20	0.117
Sn	4	S	-2	24	0.492	2.3626	6.00	0.072	5.00	2.3778	3.20	0.085	5.00	2.4017	3.15	0.192
Sn	4	Te	-2	4	0.568	2.7311	7.00	0.010	4.00	2.7661	3.59	0.086	4.00	2.7657	3.43	0.128
Sr	2	As	-3	9	0.568	2.5125	8.00	0.083	6.44	2.5296	4.02	0.089	6.44	2.7559	3.85	0.151
Sr	2	Cl	-1	2	0.573	2.2020	6.50	0.038	7.00	2.2460	3.79	0.069	7.00	2.4984	3.62	0.052
Sr	2	F	-1	36	0.446	1.8682	5.50	0.080	8.83	1.8899	3.20	0.097	8.89	1.9987	3.21	0.111
Sr	2	H	-1	4	0.470	1.8127	6.00	0.074	11.75	1.8344	3.35	0.053	11.75	2.0049	3.32	0.078
Sr	2	N	-3	7	0.544	2.0948	6.50	0.083	6.43	2.1297	3.55	0.110	6.14	2.3195	3.40	0.137
Sr	2	O	-2	85	0.455	1.9531	5.50	0.101	9.20	1.9712	3.32	0.117	9.20	2.0945	3.32	0.135
Sr	2	P	-3	8	0.663	2.3784	7.50	0.109	6.13	2.4171	4.12	0.111	6.13	2.7389	3.82	0.209
Sr	2	Sb	-3	7	0.518	2.7880	8.00	0.093	7.00	2.7953	4.19	0.094	7.00	2.9733	4.10	0.177
Sr	2	Se	-2	11	0.622	2.3672	7.00	0.075	8.00	2.3951	4.16	0.068	8.00	2.7379	3.91	0.129
Sr	2	S	-2	27	0.591	2.2677	7.00	0.098	8.15	2.2990	3.98	0.089	8.04	2.6021	3.78	0.116
Ta	2	Te	-2	4	0.593	2.2069	6.00	0.174	5.25	2.2447	3.68	0.158	5.25	2.4236	3.44	0.573
Ta	3	F	-1	5	0.555	1.5231	6.00	0.073	6.00	1.5510	2.74	0.066	6.00	1.6743	2.59	0.116
Ta	4	O	-2	2	0.546	1.7563	6.00	0.516	5.50	1.7861	2.75	0.526	5.50	1.8413	2.62	0.596
Ta	4	Se	-2	6	0.422	2.3613	6.00	0.001	6.00	2.3697	3.15	0.002	6.00	2.3908	3.20	0.002
Ta	4	S	-2	6	0.410	2.2893	5.50	0.043	5.67	2.2954	3.03	0.056	5.67	2.3083	3.10	0.123
Ta	5	Cl	-1	2	0.604	2.2144	6.50	0.023	6.00	2.2366	3.22	0.029	6.00	2.2772	3.01	0.033
Ta	5	F	-1	5	0.477	1.7901	5.50	0.031	6.40	1.8011	2.61	0.071	6.40	1.8264	2.58	0.047
Ta	5	N	-3	6	0.390	2.0412	6.00	0.047	4.67	2.0446	2.58	0.064	4.67	2.0428	2.68	0.099
Ta	5	O	-2	89	0.486	1.8682	5.50	0.113	6.09	1.8902	2.69	0.138	6.09	1.9104	2.65	0.163
Ta	5	Se	-2	3	0.654	2.5126	7.00	0.013	4.00	2.5415	3.34	0.122	4.00	2.4779	3.06	0.214
Ta	5	S	-2	6	0.622	2.2474	6.50	0.254	7.17	2.2847	3.41	0.212	7.17	2.3711	3.17	0.199
Ta	5	Te	-2	2	0.699	2.7182	7.00	0.002	4.00	2.7915	3.65	0.103	4.00	2.7178	3.30	0.195
Tb	3	Cl	-1	2	0.486	2.2953	6.00	0.013	8.50	2.3068	3.52	0.023	8.50	2.4256	3.47	0.029
Tb	3	F	-1	3	0.442	1.8404	5.50	0.013	8.67	1.8537	2.96	0.034	8.67	1.9288	2.98	0.067

Tb	3	O	-2	24	0.433	1.9568	5.50	0.064	7.83	1.9710	3.01	0.086	7.83	2.0297	3.05	0.108
Tb	3	S	-2	5	0.505	2.3638	6.00	0.117	7.60	2.3760	3.58	0.114	7.60	2.4985	3.51	0.211
Tb	4	O	-2	7	0.494	1.9624	6.00	0.072	6.00	1.9784	2.89	0.059	6.00	2.0280	2.84	0.072
Tc	4	Cl	-1	2	0.470	2.1586	6.00	0.011	6.00	2.1649	3.04	0.047	6.00	2.2040	3.02	0.044
Tc	7	O	-2	4	0.514	1.9643	6.00	0.105	4.00	1.9983	2.45	0.124	4.00	1.9173	2.37	0.174
Te	4	Br	-1	6	0.559	2.4611	7.00	0.038	6.00	2.4696	3.51	0.053	6.00	2.5395	3.35	0.111
Te	4	Cl	-1	20	0.518	2.3056	6.50	0.077	5.90	2.3240	3.27	0.110	5.85	2.3590	3.16	0.136
Te	4	I	-1	14	0.619	2.6767	7.50	0.082	6.00	2.7009	3.85	0.105	5.93	2.7796	3.59	0.127
Te	4	O	-2	106	0.401	1.9529	5.50	0.099	3.54	1.9850	2.52	0.164	3.67	1.9772	2.61	0.164
Te	6	O	-2	27	0.412	1.9134	5.50	0.139	6.00	1.9205	2.52	0.124	6.00	1.9204	2.58	0.136
Th	4	F	-1	18	0.477	1.9737	6.00	0.114	8.94	1.9889	3.06	0.087	8.94	2.0735	3.03	0.099
Th	4	O	-2	34	0.486	2.0490	6.00	0.114	8.85	2.0699	3.16	0.142	8.82	2.1569	3.11	0.197
Th	4	S	-2	3	0.500	2.5291	6.50	0.114	8.67	2.5412	3.65	0.102	8.67	2.6357	3.58	0.082
Ti	2	Br	-1	3	0.470	2.1549	6.00	0.107	6.00	2.1620	3.36	0.095	6.00	2.2713	3.34	0.117
Ti	2	Cl	-1	6	0.429	2.0343	5.50	0.043	5.67	2.0420	3.11	0.040	5.67	2.1024	3.15	0.050
Ti	3	Cl	-1	7	0.459	2.1275	6.00	0.079	6.00	2.1368	3.12	0.098	6.00	2.1982	3.12	0.127
Ti	3	F	-1	13	0.468	1.6307	5.50	0.050	6.00	1.6411	2.64	0.043	6.00	1.7086	2.63	0.056
Ti	3	O	-2	38	0.460	1.6977	5.50	0.049	6.00	1.7203	2.70	0.071	6.00	1.7822	2.70	0.087
Ti	3	S	-2	5	0.478	2.1181	6.00	0.067	6.00	2.1339	3.16	0.065	6.00	2.2083	3.13	0.085
Ti	4	As	-3	5	0.770	2.4382	7.50	0.126	4.00	2.4779	3.59	0.103	4.00	2.4777	3.14	0.213
Ti	4	Cl	-1	6	0.507	2.1258	6.00	0.089	6.00	2.1340	3.07	0.061	6.00	2.1864	3.00	0.109
Ti	4	F	-1	7	0.449	1.6617	5.50	0.034	6.00	1.6743	2.51	0.038	6.00	1.7034	2.52	0.041
Ti	4	O	-2	173	0.503	1.7239	5.50	0.093	5.99	1.7507	2.68	0.104	5.98	1.8004	2.61	0.142
Ti	4	P	-3	4	0.670	2.3749	6.50	0.094	4.00	2.3921	3.36	0.074	4.00	2.3920	3.05	0.133
Ti	4	S	-2	5	0.640	2.1745	7.00	0.198	5.00	2.2072	3.28	0.174	5.00	2.2586	3.00	0.188
Ti	4	Te	-2	3	0.716	2.4179	7.50	0.225	6.00	2.4988	3.83	0.171	6.00	2.6357	3.45	0.280
Tl	1	Br	-1	10	0.477	2.4277	6.00	0.064	8.20	2.4345	4.13	0.060	8.20	2.6526	4.09	0.066
Tl	1	Cl	-1	8	0.436	2.3679	6.00	0.050	8.38	2.3734	3.93	0.052	8.50	2.5113	3.97	0.053
Tl	1	F	-1	16	0.491	1.8274	6.00	0.054	9.63	1.8587	3.68	0.065	9.63	2.1165	3.62	0.119
Tl	1	I	-1	15	0.537	2.4869	7.00	0.037	8.07	2.4943	4.39	0.035	7.87	2.8354	4.26	0.061

Tl	1	O	-2	65	0.483	1.8902	6.00	0.086	8.02	1.9302	3.63	0.087	7.91	2.1408	3.57	0.167
Tl	1	Se	-2	31	0.486	2.3994	6.00	0.063	7.81	2.4094	4.11	0.067	7.81	2.6335	4.06	0.087
Tl	1	S	-2	88	0.455	2.3826	6.00	0.074	7.18	2.3945	3.95	0.078	7.15	2.5505	3.94	0.094
Tl	1	Te	-2	16	0.531	2.5052	6.00	0.086	6.38	2.5165	4.27	0.098	6.38	2.8031	4.15	0.099
Tl	3	Br	-1	4	0.555	2.3599	6.00	0.028	4.50	2.3780	3.41	0.086	4.50	2.4478	3.26	0.122
Tl	3	Cl	-1	9	0.514	2.2257	6.00	0.053	5.78	2.2329	3.31	0.099	5.78	2.3214	3.23	0.070
Tl	3	F	-1	4	0.413	1.8568	5.50	0.326	7.00	1.8641	2.81	0.326	7.00	1.8978	2.87	0.360
Tl	3	O	-2	18	0.338	2.0634	5.00	0.102	4.72	2.0719	2.71	0.170	5.06	2.0534	2.91	0.125
Tm	3	O	-2	34	0.421	1.9490	5.50	0.089	6.79	1.9590	2.91	0.082	6.85	1.9989	2.97	0.108
Tm	3	S	-2	16	0.516	2.3107	6.00	0.062	6.31	2.3273	3.46	0.059	6.31	2.4335	3.37	0.114
U	3	Cl	-1	8	0.494	2.3838	7.00	0.092	7.25	2.3911	3.54	0.088	7.25	2.4973	3.49	0.235
U	3	S	-2	6	0.600	2.2641	7.00	0.094	7.67	2.3003	3.73	0.113	7.67	2.5109	3.52	0.253
U	4	Cl	-1	6	0.535	2.3815	7.00	0.113	7.33	2.3942	3.49	0.105	7.33	2.4862	3.37	0.254
U	4	O	-2	10	0.428	2.0478	6.00	0.097	7.40	2.0604	2.94	0.093	7.40	2.0926	2.98	0.113
U	4	Se	-2	6	0.666	2.4430	7.50	0.124	8.33	2.4848	3.94	0.105	8.17	2.6948	3.62	0.245
U	4	S	-2	9	0.523	2.4512	7.00	0.120	7.67	2.4698	3.57	0.111	7.67	2.5656	3.47	0.212
U	5	Cl	-1	3	0.550	2.3908	7.00	0.014	6.00	2.4104	3.31	0.016	6.00	2.4395	3.17	0.027
U	5	O	-2	11	0.494	2.0114	6.00	0.130	6.55	2.0375	2.89	0.148	6.55	2.0665	2.83	0.168
U	6	Cl	-1	5	0.660	2.3859	7.00	0.104	6.00	2.4303	3.39	0.122	6.00	2.4293	3.09	0.209
U	6	O	-2	79	0.527	2.0232	6.00	0.116	7.00	2.0467	2.89	0.149	6.99	2.0396	2.76	0.238
V	2	Br	-1	2	0.483	2.1022	6.00	0.039	6.00	2.1053	3.33	0.041	6.00	2.2294	3.30	0.054
V	2	Cl	-1	4	0.442	2.0327	5.50	0.061	6.00	2.0393	3.16	0.074	6.00	2.1183	3.19	0.089
V	2	F	-1	7	0.485	1.5330	5.50	0.081	6.00	1.5539	2.79	0.071	6.00	1.6802	2.75	0.092
V	2	O	-2	4	0.477	1.5998	5.50	0.081	6.00	1.6158	2.83	0.082	6.00	1.7329	2.80	0.103
V	2	S	-2	2	0.461	1.8982	6.00	0.036	6.00	1.9162	3.09	0.038	6.00	2.0160	3.09	0.047
V	3	Cl	-1	2	0.480	2.0633	5.50	0.038	6.00	2.0716	3.10	0.081	6.00	2.1472	3.07	0.110
V	3	F	-1	13	0.448	1.6158	5.50	0.060	6.00	1.6265	2.59	0.047	6.00	1.6805	2.60	0.057
V	3	O	-2	63	0.439	1.6780	5.50	0.067	6.00	1.6959	2.64	0.070	6.00	1.7431	2.66	0.083
V	3	Se	-2	3	0.530	2.1369	6.50	0.036	6.00	2.1638	3.30	0.039	6.00	2.2728	3.19	0.049
V	3	S	-2	25	0.499	2.0262	6.50	0.047	5.92	2.0495	3.11	0.049	5.92	2.1350	3.05	0.111

V	4	O	-2	84	0.426	1.7493	5.00	0.094	5.73	1.7610	2.53	0.090	5.74	1.7749	2.57	0.089
V	4	S	-2	3	0.512	2.1676	6.50	0.004	6.00	2.1771	3.13	0.004	6.00	2.2346	3.05	0.005
V	5	O	-2	174	0.510	1.7945	5.50	0.113	4.15	1.8280	2.47	0.121	4.14	1.7991	2.39	0.189
V	5	S	-2	7	0.647	2.2925	7.00	0.061	4.00	2.3238	3.12	0.137	4.00	2.2618	2.84	0.237
W	2	Br	-1	5	0.647	1.9572	7.00	0.058	5.00	2.0190	3.55	0.050	5.00	2.2721	3.27	0.092
W	3	Cl	-1	3	0.428	2.1224	5.00	0.117	5.67	2.1293	3.02	0.080	5.67	2.1653	3.06	0.135
W	4	Br	-1	2	0.439	2.3517	6.50	0.097	6.00	2.3524	3.17	0.096	6.00	2.3803	3.19	0.113
W	4	O	-2	4	0.520	1.7456	6.00	0.060	6.00	1.8012	2.76	0.060	6.00	1.8594	2.67	0.085
W	4	S	-2	2	0.417	2.2227	6.00	0.009	6.00	2.2348	3.01	0.010	6.00	2.2539	3.07	0.012
W	5	Cl	-1	7	0.420	2.2449	6.00	0.050	6.00	2.2471	2.93	0.045	6.00	2.2555	2.99	0.055
W	5	O	-2	5	0.498	1.8197	6.00	0.054	6.00	1.8497	2.66	0.156	6.00	1.8723	2.60	0.206
W	6	Cl	-1	3	0.617	2.2150	7.00	0.082	6.00	2.2491	3.14	0.084	6.00	2.2411	2.90	0.161
W	6	O	-2	77	0.401	1.9064	5.00	0.140	5.69	1.9136	2.47	0.163	5.69	1.9103	2.55	0.155
Xe	2	F	-1	8	0.569	1.7741	6.50	0.035	2.00	2.0010	2.82	0.050	2.00	1.9955	2.66	0.076
Xe	4	F	-1	7	0.529	1.8557	6.50	0.078	4.14	1.9360	2.72	0.145	4.00	1.9345	2.60	0.193
Xe	6	F	-1	13	0.431	1.8616	6.00	0.059	5.46	1.9009	2.48	0.156	5.62	1.8913	2.53	0.141
Y	3	Br	-1	3	0.522	2.4092	6.50	0.035	6.00	2.4143	3.53	0.049	6.00	2.5184	3.44	0.068
Y	3	Cl	-1	10	0.483	2.2887	6.50	0.043	6.40	2.2930	3.36	0.047	6.40	2.3773	3.32	0.115
Y	3	F	-1	17	0.379	1.8926	5.50	0.086	7.53	1.8971	2.79	0.088	7.53	1.9053	2.91	0.090
Y	3	N	-3	7	0.620	1.9433	6.50	0.046	6.00	1.9695	3.30	0.086	6.00	2.1415	3.06	0.148
Y	3	O	-2	130	0.478	1.9038	5.50	0.140	7.28	1.9245	3.04	0.131	7.28	2.0179	3.01	0.167
Y	3	S	-2	12	0.615	2.2592	7.00	0.068	6.42	2.2990	3.66	0.081	6.42	2.4803	3.42	0.117
Yb	2	Cl	-1	2	0.409	2.2946	5.50	0.131	6.50	2.2971	3.37	0.144	6.50	2.3424	3.44	0.174
Yb	2	H	-1	4	0.496	1.5795	6.00	0.112	10.00	1.6118	3.13	0.113	10.00	1.8103	3.07	0.138
Yb	2	O	-2	2	0.510	1.6325	5.50	0.014	7.00	1.6684	3.05	0.052	7.00	1.8417	2.97	0.034
Yb	2	Se	-2	2	0.458	2.4405	7.00	0.071	6.00	2.4466	3.61	0.070	6.00	2.5432	3.61	0.086
Yb	2	S	-2	4	0.427	2.4008	6.00	0.183	7.75	2.4063	3.60	0.178	7.75	2.4826	3.65	0.227
Yb	3	F	-1	14	0.435	1.8097	5.50	0.063	7.50	1.8184	2.85	0.053	7.50	1.8768	2.88	0.068
Yb	3	O	-2	64	0.426	1.9287	5.50	0.089	6.86	1.9404	2.91	0.081	6.86	1.9856	2.95	0.092
Yb	3	Se	-2	1	0.542	2.4211	7.00	0.000	6.00	2.4336	3.59	0.000	6.00	2.5528	3.47	0.000

Yb	3	S	-2	16	0.511	2.3124	6.00	0.133	6.13	2.3253	3.43	0.136	6.13	2.4239	3.35	0.168
Zn	2	As	-3	14	0.770	1.8475	7.50	0.166	4.14	1.9698	3.64	0.109	4.14	2.2551	3.19	0.296
Zn	2	Br	-1	6	0.562	1.9874	6.00	0.026	4.00	2.0037	3.21	0.054	4.00	2.1365	3.06	0.082
Zn	2	Cl	-1	19	0.521	1.8819	6.00	0.035	4.00	1.8960	3.01	0.060	4.00	2.0005	2.92	0.083
Zn	2	F	-1	26	0.406	1.5745	5.00	0.063	5.77	1.5868	2.60	0.099	5.92	1.6230	2.69	0.085
Zn	2	I	-1	4	0.622	2.1528	6.50	0.024	4.00	2.1884	3.52	0.026	4.00	2.3625	3.28	0.042
Zn	2	O	-2	174	0.403	1.6534	5.00	0.075	4.72	1.6713	2.60	0.085	4.72	1.6986	2.68	0.086
Zn	2	P	-3	13	0.440	2.1530	7.00	0.152	3.77	2.1622	3.08	0.137	3.77	2.2044	3.10	0.157
Zn	2	Se	-2	4	0.571	2.0007	6.00	0.011	4.00	2.0451	3.27	0.031	4.00	2.1844	3.10	0.048
Zn	2	S	-2	23	0.540	1.9437	6.00	0.071	4.17	1.9782	3.16	0.071	4.17	2.1012	3.04	0.138
Zn	2	Te	-2	5	0.616	2.1513	7.00	0.082	4.00	2.1912	3.51	0.077	4.00	2.3614	3.28	0.130
Zr	2	Cl	-1	5	0.478	2.1207	6.00	0.087	5.00	2.1386	3.27	0.084	5.00	2.2362	3.24	0.104
Zr	2	H	-1	7	0.582	1.1980	6.00	0.050	8.14	1.2736	2.93	0.061	8.14	1.5702	2.75	0.130
Zr	2	I	-1	4	0.424	2.4930	5.50	0.007	6.00	2.4992	3.58	0.007	6.00	2.5579	3.63	0.009
Zr	3	I	-1	3	0.472	2.5662	6.00	0.018	6.00	2.5703	3.58	0.030	6.00	2.6409	3.56	0.038
Zr	4	Br	-1	3	0.648	2.3281	7.00	0.034	6.00	2.3608	3.56	0.016	6.00	2.4658	3.28	0.007
Zr	4	Cl	-1	7	0.608	2.1844	6.50	0.030	6.00	2.2127	3.34	0.075	6.00	2.3075	3.12	0.115
Zr	4	F	-1	48	0.388	1.8317	5.50	0.083	7.08	1.8363	2.62	0.096	7.10	1.8460	2.72	0.096
Zr	4	N	-3	4	0.570	2.0104	6.50	0.071	5.50	2.0424	3.05	0.078	5.50	2.1024	2.88	0.318
Zr	4	O	-2	85	0.490	1.8451	5.50	0.141	6.76	1.8664	2.83	0.136	6.76	1.9266	2.78	0.193
Zr	4	S	-2	6	0.626	2.2627	7.00	0.034	6.33	2.2953	3.49	0.072	6.33	2.4088	3.24	0.242

Special pseudo-atoms used in the above table:

#1 Nx^{3-} refers to the N^{3-} anion within a CN^- group;

#2 Hx^+ refers to the apparent proton position in XRD refined structures, while H^+ refers to the actual proton position as determined from structure refinements based on neutron diffraction data. The H^+ parameter should be used to locate a proton in a structure or to check neutron diffraction data, while the separate Hx^+ parameters permit the user to check whether the apparent H position in an XRD structure refinement yields the expected degree of underestimation of e.g. and O-H bond length.

#3 Nh^+ represents the ammonium cation NH_4^+ in structure determinations where the H^+ of the NH_4^+ group are not resolved.

S2. Linear regression results of fitting $R_{crystal}$ to R_{min} categorized by anion types

Table S2 Linear regression results of fitting $R_{crystal}$ (as *y*) to R_{min} (as *x*) grouped by anion types.

anion	slope	intercept	R^2
F^-	0.9949	-1.1219	0.9866
Cl^-	0.9593	-1.4841	0.9760
Br^-	0.9391	-1.5686	0.9584
I^-	0.8747	-1.5802	0.9463
O^{2-}	0.9901	-1.2046	0.9938
S^{2-}	0.9383	-1.5037	0.9750
Se^{2-}	0.9292	-1.5895	0.9487
Te^{2-}	0.9065	-1.7255	0.8996
N^{3-}	0.9058	-1.1665	0.9884
Nx^{3-} #	0.7480	-0.7078	0.9798
P^{3-}	0.8445	-1.3737	0.9390
As^{3-}	0.9147	-1.6678	0.9660
Sb^{3-}	0.9362	-1.9618	0.9800
H^-	0.8190	-0.6465	0.9615

Nx^{3-} refers to the N^{3-} anion within a CN^- group.

S3. Estimated Shannon crystal radii

Table S3 Estimated Shannon crystal radii for some ions not reported in Shannon's compilation.

ion	V_{id}	N_C	$R_{crystal}$
P	-3	-	1.777
As	-3	-	2.005
Sb	-3	-	2.341
H	-1	-	0.883
As	3	3	0.605
Au	1	2	0.710
Br	5	6	0.723
C	2	1	0.071
Co	1	2	0.527
Cu	3	4	0.618
Ga	1	9	1.677
Ga	1	8	1.504
Ge	2	2	0.549
In	1	8	1.595
Mo	2	5	0.855
N	3	1	-0.039
Nb	2	6	0.894
Nh#	1	9	1.789
Ni	3	5	0.587
Np	5	7	0.955
Np	6	7	0.890
P	3	3	0.348
Pt	3	5	0.805
Re	3	5	0.790
S	3	2	0.307
S	4	3	0.360
S	4	2	0.176
Sb	5	4	0.698
Se	4	3	0.483
Si	2	2	0.545
Sn	2	3	0.789
Sn	2	6	1.210
Ta	2	5	0.828
W	2	5	0.884
Xe	2	2	0.869
Xe	4	4	0.823
Zr	2	5	0.988
Zr	2	6	1.013
Zr	2	8	1.066
Zr	3	6	0.954

Nh^+ represents the ammonium cation NH_4^+.
